# Emerin is an effector of oncogenic KRAS-driven nuclear dynamics in pancreatic cancer

**DOI:** 10.1172/jci.insight.187799

**Published:** 2025-06-10

**Authors:** Luis F. Flores, David L. Marks, Renzo E. Vera, Ashley N. Sigafoos, Ezequiel J. Tolosa, Luciana L. Almada, David R. Pease, Merih D. Toruner, Brian Chang, Brooke R. Tader, Kayla C. LaRue-Nolan, Ryan M. Carr, Rondell P. Graham, Catherine E. Hagen, Matthew R. Brown, Aleksey V. Matveyenko, Katherine L. Wilson, David W. Dawson, Christopher L. Pin, Kyle J. Roux, Martin E. Fernandez-Zapico

**Affiliations:** 1 Division of Oncology Research,; 2 Division of Anatomic Pathology, Department of Laboratory Medicine and Pathology, and; 3 Department of Physiology and Biomedical Engineering, Mayo Clinic, Rochester, Minnesota, USA.; 4 Department of Cell Biology, The Johns Hopkins University School of Medicine, Baltimore, Maryland, USA.; 5 Department of Pathology and Laboratory Medicine, David Geffen School of Medicine, UCLA, Los Angeles, California, USA.; 6 Departments of Physiology and Pharmacology, Oncology, and Pediatrics, Schulich School of Medicine, University of Western Ontario, Verspeeten Family Cancer Centre, London, Ontario, Canada.; 7 Sanford Research, Enabling Technologies Group, Sioux Falls, South Dakota, USA.

**Keywords:** Cell biology, Oncology, Cancer

## Abstract

For over a century, scientists reported the disruption of normal nuclear shape and size in cancer. These changes have long been used as tools for diagnosis and staging of malignancies. However, to date, the mechanisms underlying these aberrant nuclear phenotypes and their biological significance remain poorly understood. Using a model of pancreatic ductal adenocarcinoma (PDAC), the major histological subtypes of pancreatic cancer, we found that oncogenic mutant KRAS reduces nuclear size. Transcriptomic and protein expression analysis of mutant KRAS–expressing PDAC cells revealed differential levels of several nuclear envelope–associated genes. Further analysis demonstrated the nuclear lamina protein, Emerin (EMD), acted downstream of KRAS to mediate nuclear size reduction in PDAC. Analysis of human PDAC samples showed that increased EMD expression associates with reduced nuclear size. Finally, in vivo genetic depletion of EMD in a mutant KRAS–driven PDAC model resulted in increased nuclear size and a reduced incidence of poorly differentiated PDAC. Thus, our data provide evidence of a potentially novel mechanism underlying nuclear size regulation and its effect in PDAC carcinogenesis.

## Abstract 2

Nuclear size/morphology are widely used as diagnostic and prognostic tools in cancer pathology. We identify the nuclear envelope protein Emerin as a nuclear size regulator downstream of mutant KRAS in pancreatic cancer

## Graphical abstract

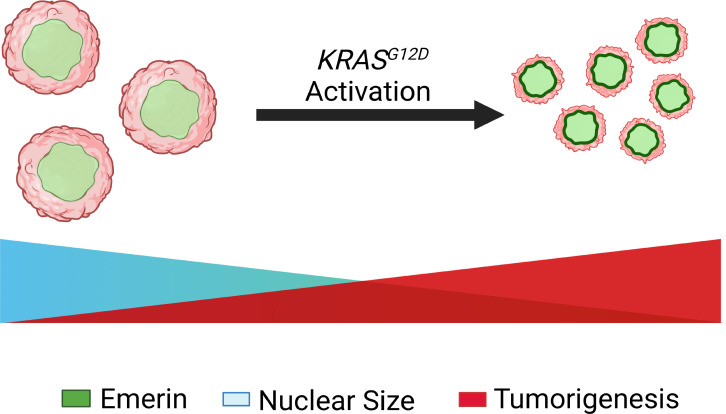

## Introduction

Since the 19th century, physicians noted abnormal nuclear phenotypes in cancer tissues. One of the most prominent irregularities observed were variations of nuclear size, a feature extensively used by modern pathologists for cancer diagnosis, staging, and prognosis (1). Furthermore, nuclear size changes correlate with lower survival in a tumor-specific manner and can affect the metastatic ability of cancer cells (2). Taken together, these findings support a functional role for nuclear size dysregulation during transformation. Investigation of nuclear size regulation has been mostly considered in the context of developmental biology. These studies yielded valuable information regarding the molecular events involved in nuclear size regulation (3–5). However, to date, the molecular events promoting nuclear size dysregulation in cancer cells and their biological consequences remain poorly understood.

Here, we define a potentially novel mechanism controlling nuclear size in pancreatic ductal adenocarcinoma (PDAC), a malignancy with significant changes in nuclear shape and size, between normal pancreas, preneoplastic lesions, and tumor tissue (6). This neoplasm is predicted to be the second cause of cancer deaths by 2040 (7), and despite significant advances in first-line therapies in the last 3 decades, the 5-year overall survival has only increased from 5% to approximately 12% (8). We report here that oncogenic KRAS (*KRAS^G12D^*), a major driver of PDAC, leads to decreased nuclear size. Transcriptomic analysis revealed several dysregulated nuclear envelope (NE) genes, and further testing identified Emerin (EMD) as an effector of *KRAS^G12D^*. EMD protein increased at the posttranscriptional level downstream of KRAS^G12D^, and depletion of EMD increased nuclear size in vitro and in vivo. Importantly, in vivo EMD depletion also led to decreased incidence of poorly differentiated PDAC. Together, our findings expand our understanding of the mechanisms controlling nuclear morphology changes involved in PDAC biology and have the potential to expand the range of therapeutic targets for this condition.

## Results

### Oncogenic KRAS reduces nuclear size in PDAC cells.

**Figure 1:**
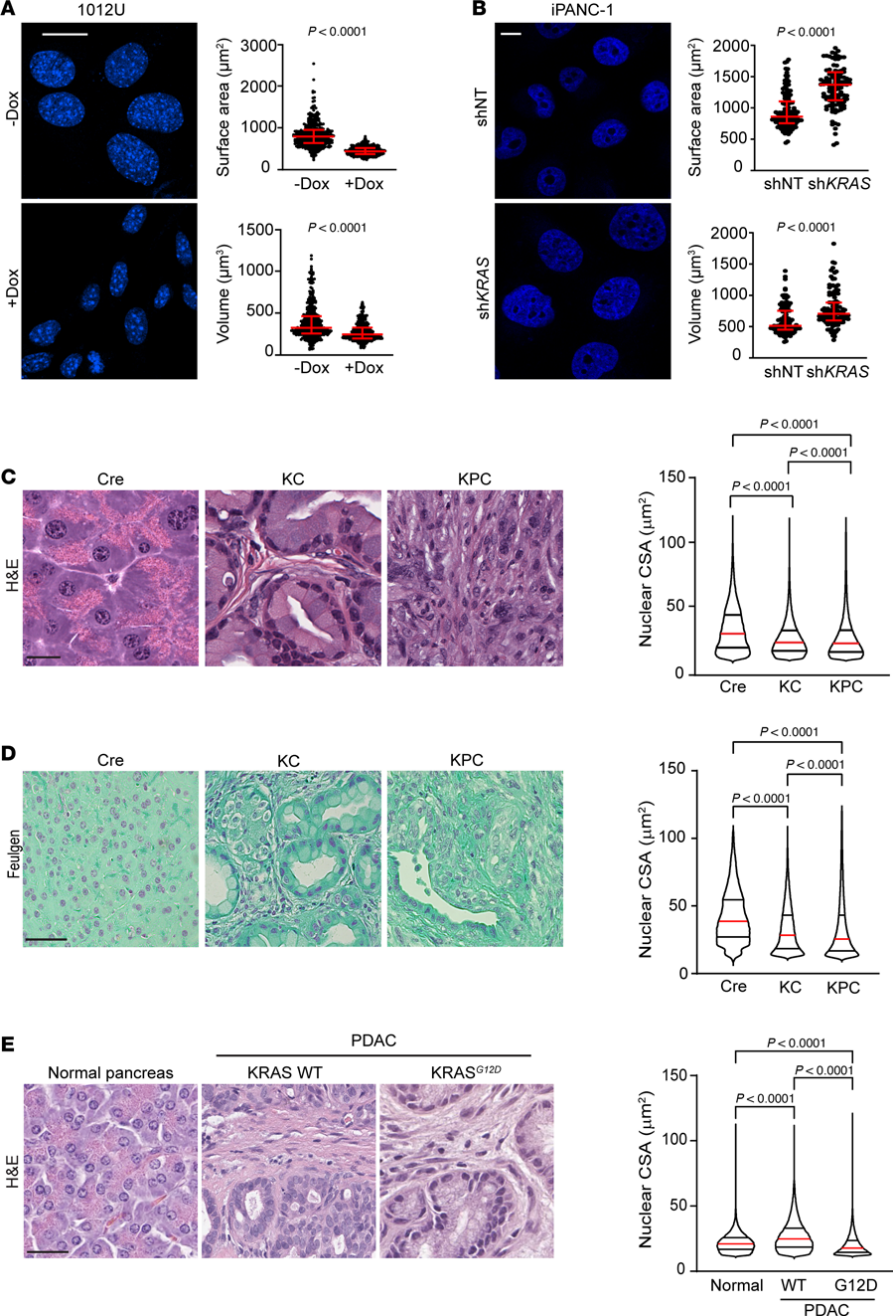
*KRAS^G12D^* induces nuclear size reduction in PDAC. (**A**) –Dox and +Dox 1012U cells (DAPI stained) with quantification of nuclear surface area and nuclear volume (–Dox *n* = 405; +Dox *n* = 383 nuclei). Scale bar: 20 μm. Scatter dot plot: median ± interquartile range. Significant difference was determined by Mann Whitney *U* test. (**B**) DAPI stained iPANC-1 cells expressing either shNT or sh*KRAS* with quantification of nuclear surface area and volume (shNT *n* = 121; sh*KRAS* *n* = 120 nuclei). Scale bar: 10 μm. Scatter dot plot: median ± interquartile range. Significant difference was determined by Mann Whitney *U* test. (**C**) H&E staining of mouse pancreas tissue with quantification of nuclear CSA (Cre *n* = 159,742; KC *n* = 500,001; KPC *n* = 589,902 nuclei). Scale bar: 25 μm. Violin plot: median ± interquartile range. Significant difference was determined by Kruskal-Wallis test, followed by Dunn’s multiple-comparison test. (**D**) Feulgen staining of Cre, KC, and KPC mice with quantification of nuclear CSA (Cre *n* = 75,697; KC *n* = 97,900; KPC *n* = 97,865 nuclei). Scale bar: 200 μm. Violin plot: median ± interquartile range. Significant difference was determined by Kruskal-Wallis test, followed by Dunn’s multiple-comparison test. (**E**) H&E stained human normal pancreata and PDAC tissues with quantification of nuclear CSA from normal, WT and mutant (*Kras^G12D^*) samples (*n* = 5/case) (Normal *n* = 56,046; *Kras* WT *n* = 89,336; *Kras^G12D^* *n* = 79,679 nuclei). Scale bar: 50 μm. Violin plot: median ± interquartile range. Significant difference was determined by Kruskal-Wallis test, followed by Dunn’s multiple-comparison test.

To define the biological significance and mechanisms underlying nuclear size dysregulation in PDAC, we initially used a mouse cell line (1012U) derived from a doxycycline-inducible (Dox-inducible) PDAC model driven by oncogenic *Kras^G12D^* — a driver mutation present in ~ 40% of human PDAC cases (9, 10). Expression of *Kras^G12D^* led to a significant reduction in median nuclear surface area and nuclear volume of doxycycline treated (+Dox) versus nontreated cells (–Dox), as observed by DAPI nuclear staining (Figure 1A). In +Dox cells, elevated KRAS signaling was confirmed by quantitative PCR (qPCR) for *Kras* expression and by Western blots for the levels of phosphorylated ERK (pERK), a known downstream effector of KRAS and established marker for this activity of this GTPase (11) (Supplemental Figure 1A; supplemental material available online with this article; https://doi.org/10.1172/jci.insight.187799DS1). These results were validated in a second doxycycline-inducible *Kras^G12D^* mouse cell line (4292F) (Supplemental Figure 1B).

To assess the dependency on KRAS for the nuclear size changes, we first investigated if the effect of mutant KRAS can be reversed. We grew 1012U cells in a sequential fashion in –Dox media for 72 hours (basal state) and then in +Dox media for 72 hours (induced state), followed by –Dox media for 72 hours (deinduced state). Analysis of DAPI-stained nuclei established that KRAS-driven reduction in nuclear size was reversible upon deinduction of *KRAS^G12D^* (Supplemental Figure 2, A and B). Changes in *Kras* expression and activity were confirmed by qPCR and the levels of pERK, respectively (Supplemental Figure 2, C and D). We then evaluated the effect on nuclear size and surface in human PDAC cells (inducible PANC-1 [iPANC-1]) containing a *KRAS^G12D^* mutation. In this system, doxycycline induces a shRNA against oncogenic *KRAS* or a shNT (nontargeting) control (Figure 1B and Supplemental Figure 1C). A second cell line was also tested using PANC-1 cells by transfecting with either *KRAS* or NT siRNAs (Supplemental Figure 1D). These results were confirmed in a third mutant KRAS line, nestin^+^ epithelial (HPNE) cells stably expressing the second most common PDAC mutation *KRAS^G12V^* (HPNE-p16shRNA-*KRAS^G12V^*) (9, 10), which demonstrated that knockdown of KRAS resulted in an increased nuclear size (Supplemental Figure 1E). Together, all these experiments demonstrate the requirement of mutant KRAS in the regulation of nuclear size.

To determine if other KRAS mutations can drive similar nuclear phenotype, nuclear size was examined in PDAC lines carrying *KRAS^G12C^* mutation (MiaPaCa-2), and in WT lines for KRAS (BxPC3, Hs766T and HPNE) and HPNE-p16shRNA-*KRAS^G12V^*. Nuclear volume and surface area were measured, and results revealed that when compared with the normal pancreatic line, HPNE, both WT KRAS cell lines (BxPC3 and Hs766T) were comparable (not significant) in nuclear size. All KRAS mutant lines containing either G12V, G12C, or G12D (PANC-1) mutations were significantly decreased in nuclear volume and surface area compared with normal or WT KRAS (Supplemental Figure 3, A and B). Based on the above results, we sought to define if disrupting the MEK/ERK signaling, the most common downstream effector of all *KRAS* mutants (12), affects nuclear size changes. The 1012U cells induced (+Dox) for 48 hours were treated with vehicle (DMSO) or MEKi (U0126) and imaged for immunofluorescence (IF) analysis and Western blot 24 hours after treatment. IF images showed an increase of both nuclear volume and surface area in the MEKi-treated cells compared with vehicle. Western blot analysis confirmed the efficacy of the MEKi on the pERK (Supplemental Figure 4, A–C). These data demonstrate that any mutation on KRAS resulted in reduced cell size and that inhibiting the MEK/ERK signaling cascade prevented the decreased nuclear size that was observed in PDAC cells with intact MEK/ERK signaling.

One feature of *KRAS^G12D^* is the activation of mitogenic signaling, increasing the rate of cell proliferation (9, 10). It has been reported that changes in cell proliferation could affect nuclear (and overall cellular) size. Therefore, to further understand the effects on nuclear size regulation common to other PDAC mitogenic signaling, we transfected *cMYC* and constitutively nuclear *CYCLIN D1^T286A^* in –Dox 1012U cells (13, 14). Interestingly, overexpression of these oncogenes did not alter nuclear size (Supplemental Figure 5, A and B) but did promote cell proliferation (Supplemental Figure 5C). Expression control and localization of *cMYC* or nuclear *CYCLIN D1^T268A^* were performed by Western blotting and IF, respectively. Results confirmed similar expression of both *cMYC* and *CYCLIN D1^T268A^* in –Dox 1012U cells (Supplemental Figure 5D) and nuclear localization (Supplemental Figure 6A). These findings show that the nuclear size changes induced by *KRAS^G12D^* are not common to other pathways driving PDAC cell growth. Cell crowding can affect cellular size and morphology; therefore, we aimed to define this variable by measuring the Delaunay internuclear distance (Supplemental Figure 7, A and B) and corrected for differences in nuclear size by taking the internuclear distance divided by the nuclear diameter. Our results reveal no statistical differences between –/+Dox condition (1012U) or HPNE versus HPNE-p16shRNA-*KRAS^G12V^*, indicating comparable crowding under both conditions (Supplemental Figure 7, A and B). Based on these findings, we evaluated the effect of mutant KRAS on cytoplasmic size; to this end, 1012U cells in –/+Dox condition were stained with DAPI (nuclear) and ViaFluor (cytoplasmic) (Supplemental Figure 8A). Nuclear and cytoplasmic volume were measured by imaging analysis and revealed that, in +Dox condition, both were statistically reduced compared with –Dox condition, indicating that the overall size of the cell was reduced in mutant KRAS expression (Supplemental Figure 8B).

### Mouse and human Mutant KRAS^G12D^ PDAC associates with smaller nuclear size.

To determine if similar nuclear size changes are present in pancreatic cancer in vivo, we used 2 independent mouse PDAC models, exocrine-pancreas–specific Cre-activated mutant *KRAS^G12D^* (KC) and exocrine-pancreas–specific Cre-activated *KRAS^G12D^* in combination with deletion of *Tp53* (KPC) (15, 16) (Supplemental Figure 9, A and B). Normal and PDAC tissues from these models were IHC stained for pERK to confirm the activation of the downstream effector of KRAS (Supplemental Figure 10A) and H&E stained to analyze nuclear size (Figure 1C). Analysis of nuclear cross-sectional area (CSA) using QuPath quantitative pathology software demonstrated reduced median nuclear CSA in tumor samples versus normal exocrine cells (Cre control) in both models (Figure 1C). Next, we evaluate if KRAS also induces nuclear changes in the most common preneoplastic conditions, the pancreatic intraepithelial neoplasia (PanIN) in KC mice, a model with a high incidence of PanIN. Nuclear CSA analysis showed PanIN’s reduced nuclear size compared with WT (Cre) mice and yet had larger nuclei compared with tumor (Supplemental Figure 11, A and B). These results indicate that, while oncogenic KRAS does affect PanIN nuclear size, lesions of PDAC still have a greater nuclear size reduction. Nuclear CSA quantification was further validated utilizing Feulgen staining and ImagePro quantitative software in Cre, KC, and KPC tissues in which we confirmed the same decrease in nuclear size in oncogenic KRAS samples as seen previously (Figure 1D). Finally, we assessed the relevance to human PDAC from KRAS mutant specimens containing tumor versus adjacent normal tissues. Consistent with observations in mouse PDAC, quantitative analysis of nuclear CSA revealed that nuclei from human mutant KRAS pancreatic tumor tissue were reduced in size compared with adjacent normal or KRAS WT PDACs (Figure 1E). IHC validation of pERK activation was done, and interestingly, while there was a range of pERK activation of WT KRAS and mutant KRAS, overall pERK expression was higher in mutant KRAS PDAC cases (Supplemental Figure 10B). We conclude that oncogenic KRAS expression decreases nuclear size in PDAC models, and our evidence suggests that decreased nuclear size in human PDAC may also be a consequence of oncogenic KRAS signaling.

### EMD is an effector of oncogenic KRAS to control nuclear size.

**Figure 2:**
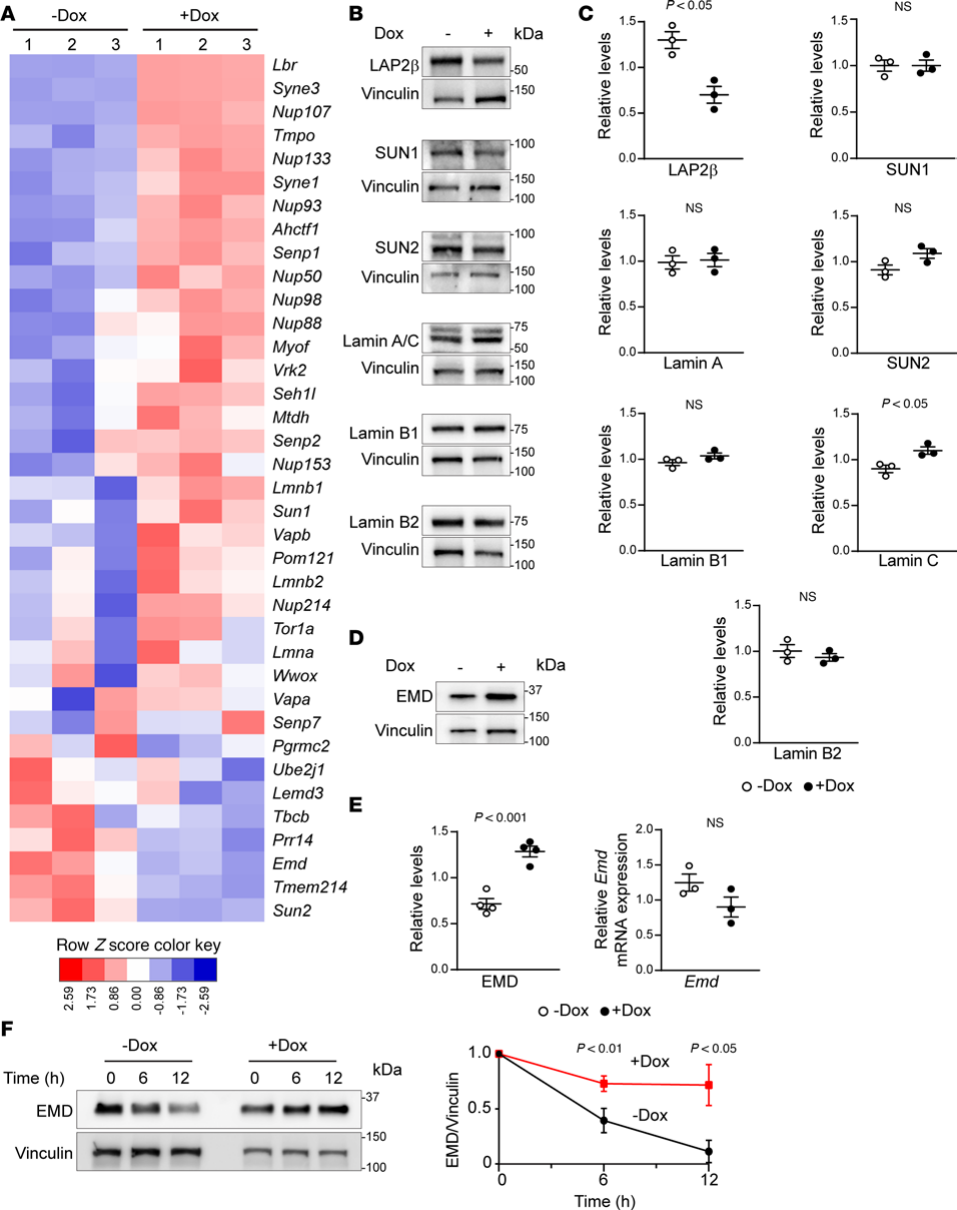
EMD protein levels are stabilized by oncogenic KRAS. (**A**) RNA-Seq heatmap of nuclear envelope genes from –Dox and +Dox 1012U cells (*n* = 3/group) 72 hours after doxycycline treatment. (**B**) Western blot screen of nuclear envelope proteins in 1012U cell under –/+Dox condition. (**C**) Densitometry for nuclear envelope proteins normalized to Vinculin (*n* = 3/group). Scatter dot plot: mean ± SEM. Significant difference was determined by Student’s *t* test. (**D**) Western blot of EMD protein with Vinculin loading control in 1012U –/+Dox condition. (**E**) Densitometry for EMD protein levels normalized to Vinculin and qPCR of *EMD* gene expression relative to *mPrt/Tbp* (*n* ≥ 3). Scatter dot plot: mean ± SEM. Significant difference was determined by Student’s *t* test (protein expression) and Mann Whitney *U* test (mRNA expression). (**F**) Cycloheximide assay for 0-, 6-, and 12-hour time points in 1012U –/+Dox condition followed by EMD Western blot with densitometry quantification relative to Vinculin (*n* = 3/group). Line plot: mean ± SEM. Significant difference at each time point was determined by Student’s *t* test.

Alterations of NE protein levels play a role in nuclear size regulation under normal and disease conditions (3–5, 17–19). Therefore, we examined changes of widely known components of the NE (20) upon induction of *KRAS^G12D^*. RNA-Seq analysis showed dysregulation of several genes encoding NE proteins following *KRAS^G12D^* induction in 1012U line (Figure 2A). We confirmed RNA-Seq changes by Western blotting for select, well-known NE proteins ranging from highly expressed mRNAs in mutant *KRAS* expressing cells (1012U +Dox) — i.e., LAP2β (gene name *Tmpo*) — to lowly expressed mRNAs — i.e., EMD and SUN2 (Figure 2, B and D). Densitometry analysis was performed to determine protein levels relative to vinculin loading control (Figure 2, C and E). Notably, EMD protein levels were significantly increased in *KRAS^G12D^*-expressing cells despite a lack of increase in its mRNA expression (Figure 2, D and E). These results were further validated in a second inducible mouse and human models, 4292F and iPANC-1, respectively. Western blot and its corresponding densitometry showed an increase of relative EMD protein levels in 4292F under mutant KRAS (+Dox) condition. Conversely, knockdown of KRAS in iPANC-1 cells lowered the levels of EMD (Supplemental Figure 12, A and B). EMD is an interesting candidate, as it is canonically an inner NE transmembrane protein, with a wide range of roles including tethering chromatin to the nuclear periphery, acting as an effector of mechanotransduction activity, and functioning as a structural component of the nucleus (21–24). EMD has also been implicated in metastatic potential of other cancer types (25–27). EMD had a significant increase in protein levels in *KRAS^G12D^*-induced cells, without a significant increase in mRNA levels (Figure 2E), suggesting alterations in protein stability. We postulated that increased EMD protein levels in *KRAS^G12D^*-expressing cells were due to an enhanced protein half-life. Therefore, we performed a cycloheximide chase assay and confirmed that EMD’s half-life was increased in *KRAS^G12D^*-induced cells (Figure 2F).

**Figure 3:**
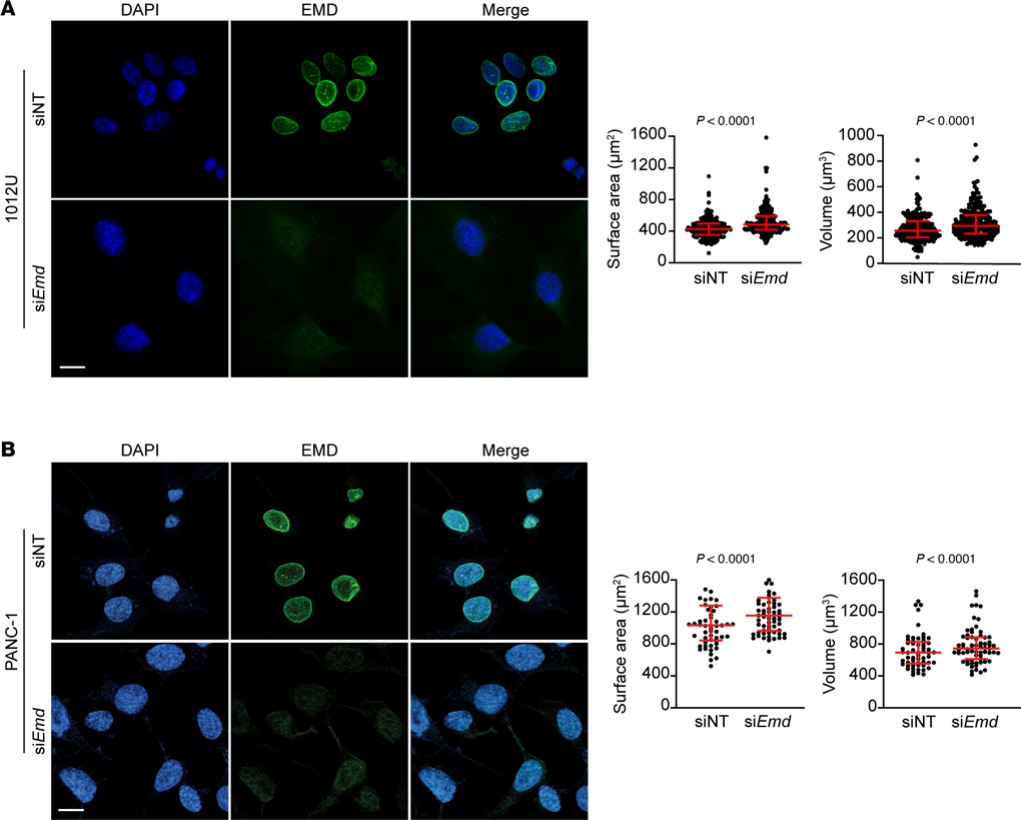
EMD is required by *KRAS^G12D^* to regulate nuclear size. (**A**) IF of +Dox condition 1012U cells transfected with siNT and si*EMD*, with quantification of nuclear surface area and volume (siNT *n* = 226; si*EMD* *n* = 233 nuclei). (**B**) Nuclear surface and volume quantification (siNT *n* = 143; si*EMD* *n* = 115 nuclei) of PANC-1 cells transfected with siNT and si*EMD*. (**A** and **B**) Scale bars: 10 μm. Scatter dot plot: median ± interquartile range. Significant difference was determined by Mann Whitney *U* test.

Having identified EMD as candidate effector of KRAS, we tested its ability to regulate nuclear size. *EMD* depletion by small interfering-RNA (siRNA) (shown by IF) led to increased median nuclear surface area and volume in both *KRAS^G12D^* mutant induced murine 1012U (+Dox) cells and in human PANC-1 cells carrying the same mutant *KRAS* (Figure 3, A and B). Increase in nuclear size due to EMD depletion in +Dox 1012U cells was validated using an independent *EMD* siRNA pool (Supplemental Figure 13A). Thus, we identified EMD as a NE protein increased upon *KRAS^G12D^* induction and showed that its depletion suppressed nuclear size reduction in *KRAS^G12D^*-expressing cells (1012U +Dox), suggesting that EMD is a candidate effector of *KRAS^G12D^* induced changes.

To gain further insight into the specificity of nuclear size regulation by EMD, we performed siRNA-mediated depletion of additional NE proteins, *Lamin A/C* and *Lamin B1*, in *KRAS^G12D^*-induced cells (1012U +Dox). Interestingly, nuclear size was not affected by *Lamin A/C* or *Lamin B1* depletion (Supplemental Figure 14, A and B). We suspect that lamin depletion does not affect nuclear size as EMD does for a number of reasons. First, EMD is distinct from lamins by having its own unique set of binding partners. Second, A- and B-type lamins may compensate for one another, masking the effects of knockdowns. Third, EMD is known to localize to various compartments outside the nucleus such as the outer nuclear membrane and endoplasmic reticulum, providing broader ways to affect cell biology. Finally, neither A- or B-type lamin protein levels change in oncogenic KRAS–expressing PDAC cells, suggesting that lamin protein levels do not contribute to the nuclear phenotype produced by oncogenic KRAS signaling.

We then performed line scans to determine the localization of EMD with and without *KRAS^G12D^* in 1012U cells. Results confirmed increased enrichment of EMD to nuclear periphery under +Dox versus –Dox condition with 72% and 52%, respectively (Supplemental Figure 15, A and B). Additional IF was performed to determine if depletion of *EMD* affects cellular structural components such as α-tubulin and centrin (Supplemental Figure 16, A and B) or phalloidin and cytokeratins (Supplemental Figure 17, A and B) as markers of cilia, microtubules, cytoskeleton, and epithelial cells, respectively. Images indicated that 1012U +Dox under siNT or si*EMD* treatment appeared similar in localization, organization, and expression in both conditions.

### EMD modulates KRAS-induced pancreatic carcinogenesis.

Next, to determine if EMD could regulate KRAS oncogenic function, we knocked down EMD and performed proliferation assays on 2 different cell lines. We found that, upon EMD knockdown in both mouse and human cell lines (1012U +Dox and HPNE-p16shRNA-*KRAS^G12V^*), there is a statically significant decrease in proliferation at both 48 and 72 hours (Supplemental Figure 18A). Western blots were performed and confirmed protein reduction of EMD at 48- and 72-hour time points (Supplemental Figure 18B). These findings prompted us to study the in vivo role of EMD in PDAC biology and nuclear size regulation. To accomplish this, we generated exocrine pancreas–specific *Emd*-KO (*Emd^+/–^* or *Emd^–/–^*) mice using the established pancreas-specific p48-Cre-lox recombination mouse model Cre-*Emd*^+/–^ or *Emd*^–/–^ (CE) (Supplemental Figure 19A). Proper mice recombination and decreased *Emd* expression were confirmed via PCR and qPCR, respectively (Supplemental Figure 19B). Due to *Emd* being a x-linked gene, all heterozygous mice for *Emd* are female. Previous work involving mice lacking Emd demonstrated that there were no overt phenotypes (23, 28), and as expected, Cre and CE mice were viable and born at the expected Mendelian ratio. Tissue analysis of pancreas from Cre control mice and CE mice demonstrated normal pancreatic development (Supplemental Figure 19D). Furthermore, we examined pancreatic markers of differentiation and function by IF and observed similar staining patterns of amylase (exocrine pancreas), insulin, and glucagon (endocrine pancreas) expression and showed comparable percent β cell area between Cre and CE mice (Supplemental Figure 19, C and E). Thus, we conclude that animals with *Emd* loss do not present apparent developmental abnormalities and have pancreata within normal limits similar to WT mice.

**Figure 4:**
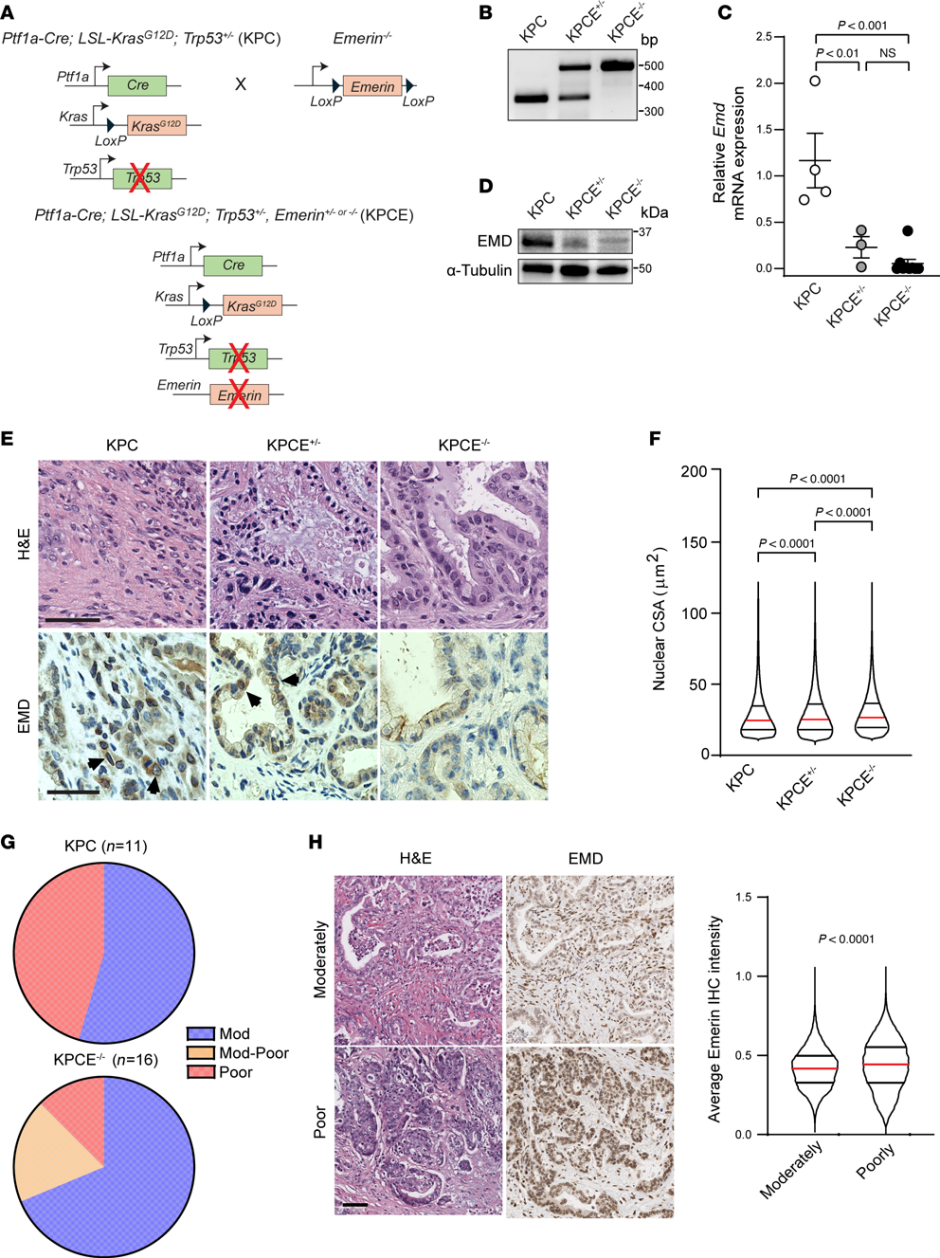
EMD is an effector of nuclear size change downstream of oncogenic KRAS in vivo. (**A**) Schematic representing animal crosses to generate *Ptf1a-Cre, LSL-Kras^G12D^, Trp53*^+/–^*, EMD^+/–^,* or *EMD^–/–^* (KPCE) mice using *Ptf1a-Cre, LSL-Kras^G12D^, or Trp53*^+/–^ (KPC) with *EMD*^+/–^ or *EMD*^–/–^ mice. (**B**) Pancreatic tissue confirmation of EMD recombination. WT = 349 bp, *Emd*^+/–^ = 349/490 bp, *Emd*^–/–^ = 490 bp. (**C**) qPCR of *Emd* expression relative to *mPrt/Tbp* in pancreata from KPC (*n* = 4), KPCE^+/–^ (*n* = 3), and KPCE^–/–^ (*n* = 9) mice**.** Scatter dot plot: mean ± SEM. Significant difference was determined by ANOVA, followed by Tukey’s multiple-comparison test. (**D**) Western blot of mice pancreas for EMD protein level with α-tubulin loading control. (**E**) H&E-stained pancreatic tumors from KPC, KPCE^+/–^, and KPCE^–/–^ mice. Scale bar: 50 μm. IHC staining of EMD; arrow heads indicate positive EMD expression. Scale bar: 200 μm. (**F**) Quantification of nuclear CSA from H&E-stained pancreatic tumors from KPC, KPCE^+/–,^, and KPCE^–/–^ mice (KPC *n* = 456,858 nuclei; KPCE^+/–^ *n* = 572,027 nuclei; KPCE^–/–^ *n* = 451,757 nuclei). Violin plot: median ± interquartile range. Significant difference was determined by Kruskal-Wallis test, followed by Dunn’s multiple-comparison test. (**G**) Histopathological analysis of KPC (*n* = 11) and KPCE^–/–^ (*n* = 16) pancreatic tumors. (**H**) H&E and IHC staining of EMD in moderately and poorly differentiated human PDAC samples, and quantification of EMD intensity by grade. Moderately differentiated PDAC nuclei = 47,963 from *n* = 22 cases, poorly differentiated PDAC nuclei = 50,056 nuclei from *n* = 15 cases. Scale bar: 80 μm. Violin plot: median ± interquartile range. Significant difference was determined by Mann Whitney *U* test.

We generated KPC and KPC with *Emd^–/–^* KO (KPCE) mice (Figure 4A) to investigate how Emd affected nuclear size and PDAC biology in vivo. A mouse model was confirmed with proper recombination, decreased mRNA expression, and loss of protein levels for EMD in mouse pancreas tissue (Figure 4, B–D). Tumor incidence between the 3 genotypes was similar, with a slight increase of tumor incidence in the KPCE^–/–^ cohort (89%) versus KPC and KPCE^+/–^ (71% and 70%, respectively) cohorts (Supplemental Figure 20A). Additionally, tumor weight, body weight, and percent tumor were not observed to be different among the 3 genotypes (*P* = 0.64, 0.57, and 0.60) (Supplemental Figure 20B). Kaplan-Meier analysis of overall survival showed no significant difference (*P* = 0.3028) between KPC, KPCE^+/–^, or KPCE^–/–^, with median survival being 209, 199, and 175 days, respectively (Supplemental Figure 20C). IHC was performed to demonstrate the reduction of EMD in KPCE^+/–^ and KPCE^–/–^ compared with the KPC group (Figure 4E). Next, utilizing whole-slide imaging, we quantified nuclear CSA in H&E images of pancreatic tumors and observed that *Emd* depletion led to increased nuclear size in vivo (Figure 4, E and F). Similar to the in vitro models, we determine the Delaunay internuclear distances in H&E images that were used to measure nuclear CSA from KC, KPC, KPCE^+/–^, or KPCE^–/–^ (Supplemental Figure 21A). When using the Delaunay internuclear distance divided by the diameter of nucleus, to correct for nuclear size differences, results showed no statistical difference in crowding of KC, KPC, KPCE^+/–^, and KPCE^–/–^ (Supplemental Figure 21B). *Emd* depletion in PDAC tissue also led to an increased percentage of mice with moderately differentiated tumors, which is associated with a more positive outcome in PDAC (Figure 4G). Finally, we examined the expression of EMD in human PDAC and its association with tumor grade. In agreement with the mouse model results, poorly differentiated tumors showed higher levels of EMD (Figure 4H).

It is generally accepted that nuclei become enlarged at initial stages of PDAC development; however, to our knowledge, there has not been a thorough analysis of nuclear size in PDAC cell lines and primary tissue (29). Our observations show that a reduced nuclear size is specific to oncogenic *Kras* and not *cMyc* or *Cyclin D1^T286A^* expression in PDAC cells; this may partially explain why cancer types reliant on distinct oncogenic signaling networks have an increase in nuclear size, while others have a decreased nuclear size. Considering our findings in the current paradigm of PDAC nuclear size, we propose that nuclear size dysregulation in PDAC may result in some nuclei that are larger than those of normal tissue; however, at the population level, there is a significant shift toward smaller nuclei, which can be attributed to robust oncogenic KRAS signaling.

## Discussion

PDAC nuclear pleiomorphisms including overlapping nuclei, irregular chromatin distribution, changes in N/C ratios, and nuclear size have been extensively reported in PDAC. These general nuclear features of PDAC are not unique to this cancer as many cancers present abnormal nuclear phenotypes. Interestingly, as we identified EMD to mediate nuclear size reduction in PDAC cells downstream of mutant oncogenic KRAS, another study involving breast cancer found that EMD was causal in nuclear size enlargement (30). Breast cancer, which is often driven by different oncogenes such as HER2 and estrogen receptor (ER) pathways presents many nuclear abnormalities that include irregular nuclear shape, enlarged nuclear size, increased nuclear to cytoplasmic ratio, and nuclear invaginations. These nuclear pleiomorphisms are graded and correlate with aggressiveness and prognosis. In Liddane et al. (30), the authors reported that EMD was reduced in invasive breast cancer cells compared with normal cells and that EMD loss was causal to a reduced nuclear size. Furthermore, restoring EMD expression was shown to inhibit migration, supporting their model in which EMD plays a central role in metastatic transformation in breast cancer. Others have shown that aggressive metastatic breast cancer is characterized by enlarged nuclei, and there is a correlation between antiestrogen therapy treatment of preoperative breast cancer with a reduction of nuclear size in tumors (31). Thus, these studies highlight how EMD could have contrasting phenotype and effect in distinct cancer cell types. However, this divergence is not completely unexpected. Breast cancer and PDAC are driven by different oncogenic networks, resulting in distinct neoplastic features. These differences ultimately lead to comparatively unique molecular compositions of the NE. Furthermore, NE proteins have complex interaction networks with other NE proteins, transcription factors, structural proteins, and chromatin (21, 32, 33). Altogether, these aspects limit our ability to predict the biological implications of a NE protein in distinct contexts. Our study causally links nuclear size and EMD in PDAC biology, but we have yet to identify the exact biochemical/molecular mechanisms of the contribution of nuclear size regulation to PDAC. More in-depth studies will be required to understand these processes; nonetheless, continuing to highlight the NE’s role in PDAC may lead to development of novel biomarkers or therapeutic targets, as these proteins have many relevant implications in PDAC biology ranging from gene expression to cellular structure.

## Methods

### Sex as a biological variable.

Our study examined male and female animals, and similar findings are reported for both sexes except for data involving KPCE^+/–^ mice, which are all female due to *Emd* being an X-linked gene.

### Breeding and genotyping.

Control animals p48-cre (34), 1XJ6 B6.129*-* *LSL-Kras^G12D^* (34), and *Tp53^fl/+^* (15) were previously described (16). C57BL/6 *EMD*-deficient (*Emd^–/–^*) mice were obtained a gift from Jan Lammerding (Cornell Research facility, Cornell University) (23). Groups of mice were crossed to create the following cohorts: (a) *p48-cre* (Cre); (b) *p48-cre* *Emd^+/–^* or *Emd^–/–^* (CE); (c) *LSL-Kras^G12D^* *p48-cre* (KC); (d) *LSL-Kras^G12D^* *p48-cre* *Tp53^fl/+^* (KPC); and (e) *LSL-Kras^G12D^* *p48-cre* *Tp53^fl/+^* *Emd^+/–^* or *Emd ^–/–^* (KPCE). Allele-specific PCR was used to verify KO of *Emd*. With *Emd* being a X-linked gene, only female mice can be heterozygous or homozygous KO for *Emd,* while male mice could only be WT or homozygous KO. Common reverse 5′-CCCAGCTCCTATCCCAGTAGGA-3′, WT forward 5′-TCTAGTTGGGTGCAAGGTCTAGC-3′, and floxed allele forward 5′- TTGTCTGCCATGGACGACTATGC-3′. Primers were obtained from IDT (WT = 349 bp, *Emd^+/–^* = 490/349 bp, *Emd^–/–^* = 490 bp).

### Plasmids.

Empty vector, pcDNA-HA2 was a gift from Scott Kaufmann (Division of Oncology Research, Department of Oncology, Mayo Clinic). pcDNA-*CYCLIN D1*-T286A-HA was a gift from Bruce Zetter (Vascular Biology and Department of Surgery, Children’s Hospital, Harvard Medical School, Boston, Massachusetts, USA) (RRID: Addgene 11182) (35). *cMyc* plasmid, pCDNA3-HA-HA-human*CMYC*, was a gift from Martine Roussel (Department of Tumor Cell Biology, St. Jude Children’s Research Hospital, Memphis, Tennessee, USA) (RRID: Addgene 74164) (36).

### Western blot.

Cells were lysed in modified RIPA buffer (50 mM Tris-HCl [pH 7.5] [Invitrogen, 15506-017], 1.0% NP-40 [Fluka BioChemika ,74385], 150 mM NaCl [Sigma-Aldrich, S9625], 5 mM EDTA [Life Technologies, AM9261], 0.25% sodium deoxycholate detergent [Sigma-Aldrich, D6750], and 1.0% SDS [Bio-Rad, 239753]) supplemented with phosphatase inhibitors (Thermo Fisher Scientific, PI78420) and cOmplete protease inhibitor cocktail (Roche, 11836145001). Lysate was sonicated on ice at 10% frequency for 3 seconds for 5 cycles with 3 seconds between each cycle. Quantification of the protein was performed using a BCA-based kit (Thermo Fisher Scientific, 23227) with a BSA standard curve. Equal amounts of protein were resolved by SDS-polyacrylamide gel electrophoresis; the proteins were then transferred to PVDF membranes (EMD Millipore, IPVH00010). Membranes were blocked with 3% BSA (Sigma-Aldrich, A8806) in TBST for 1 hour at room temperature and then incubated in primary antibody overnight at 4°C on a rocker platform. Membranes were washed 3× (20 minutes) with 1× TBST before being probed with secondary antibody for 1 hour at room temperature and washed 3× (20 minutes) with 1× TBST. Blotted membranes were developed with SuperSignal ECL detection kit (Thermo Fisher Scientific, 34094) and imaged with Bio-Rad ChemiDocTM and ChemiDoc MP Imaging System. Densitometry quantification was performed using ImageJ (v.1.49p; NIH) software by measuring the AUC for the band intensity in each lane; protein levels were measured relative to loading control. Refer to Supplemental Table 1 for antibody details.

### qPCR.

Total RNA was extracted from cells using TRIzol reagent (Invitrogen, 15596018). Total RNA (2 μg) was reverse transcribed to cDNA using the High-Capacity cDNA kit (Applied Biosystems, 4368814). The resultant cDNA was amplified by PCR using a quantitative method. Specific genes were amplified using the PerfeCTa SYBR Green SuperMix (Quanta, 95054-500) and Bio-Rad CFX384 Real Time PCR instruments with validated primers specific for the genes of interest. Amplifications were carried out as follows: 10 minutes at 95°C, 40 cycles of 15 seconds at 95°C, and 1 minute at 60°C. Expression primers used are: *EMD* (mouse) forward: 5′-TCGTCATCTTCTTCATTCTCCTATC-3′, reverse: 5′-TCATTATAGTCCTTGCTCTGGTAAA-3′; forward: 5′- ctgtttgttgtcaccttttcag-3′, reverse: 5′-cccagctcctatcccagtagga-3′; *Kras* (mouse) forward: 5′-AGAGGACTCCTACAGGAAACA-3′, reverse: 5′-GTCCCTCATTGCACTGTACTC-3′; *Prt* (mouse) forward: 5′-AAGTGTTTATTCCTCATGGACTGA-3′, reverse: 5′-CTCCCATCTCCTTCATGACATC-3′; *Tbp* (mouse) forward: 5′-GAAGTTCCCTATAAGGCTGGAAG-3′, reverse: 5′-AGGAGAACAATTCTGGGTTTGA-3′; *KRAS* (human) forward: 5′-ACACAAAACAGGCTCAGGACT-3′, reverse: 5′-ACACCCTGTCTTGTCTTTGCT-3′; *HPRT* (human) forward: 5′- CCTGGCGTCGTGATTAGTGAT-3′, reverse: 5′- AGACGTTCAGTCCTGTCCATAA-3′; and *TBP* (human) forward: 5′- TATAATCCCAAGCGGTTTGC-3′, reverse: 5′-CCCAACTTCTGTACAACTCTAGCA-3′. Primers were obtained from IDT.

### Cell proliferation.

CyQUANT cell proliferation assays were performed by seeding cells in a 96-well plate at 2,000 cells per well. CyQUANT Cell Proliferation Assay Kit (Thermo Fisher Scientific, C7026) was used according to manufacturer’s guidelines. At the indicated times, cells were lysed using CyQUANT dye mix, and total cellular nucleic acid was measured by fluorometer at 508/527 nm wavelengths.

### Line scan.

The 1012U cells in the presence and absence of doxycycline stained with EMD were used for line scan analysis. *Z* stack confocal images were taken, and the middle *z* plane was used for analysis. Line scan analysis was performed using ImageJ Fiji. A measurement line was drawn through the long axis of each nucleus. The percent of EMD at the periphery was calculated by taking the sum of the signal within 3 μm from the edges of the nucleus divided by the total signal across the line.

### IF.

Indirect IF was performed on cells seeded on round 25 mm coverslips in a 6-well plate (Thermo Fisher Scientific, 174985). Cells were washed 3× with 1× PBS and fixed for 15 minutes with 3.2% (vol/vol) paraformaldehyde (Thermo Fisher Scientific, 043368.9M) in PBS at room temperature. For centrin or OSCAR/cytokeratin staining, cells were fixed with ice-cold methanol for 15 minutes on ice; no further permeabilization was performed in this case. Cells were washed again 2× with 1× PBS and then permeabilized in 0.1% Triton X-100 (MilliporeSigma, T8787-50ML) for 6–10 minutes (depending on confluency) on ice. Cells were then blocked for 1 hour at room temperature with IF blocking buffer (5% normal goat serum, 5% glycerol, and 0.05% sodium azide in PBS) and then incubated with primary antibody for 1 hour at room temperature. Following primary antibody incubation, cells were washed 3× with 1× PBS before being incubated with secondary antibodies for 1 hour at room temperature and washed 3× with 1× PBS. Coverslips were mounted with Prolong gold with DAPI (Life Technologies, P36931) and aged overnight. Images were obtained at 10×–40× magnification using Zeiss Axio Observer Z1 microscope using ZenPro software. Refer to Supplemental Table 1 for antibody details.

### Cycloheximide chase assay.

1012U cells were cultured for 48 hours with or without doxycycline (MilliporeSigma, D9891). At 48 hours, cells were treated with 30 μg/mL cycloheximide (MilliporeSigma, 01810-1G) and harvested at 0, 6, and 12 hours. After each time point, cells were collected, and cell lysates were prepared for Western blot as described previously (*n* = 3). Refer to Supplemental Table 1 for antibody details.

### RNA-Seq.

RNA-Seq was performed in 1012U cells under –Dox and +Dox conditions. Total RNA was isolated 72 hours after doxycycline treatment using TRIzol and further purified with a RNeasy Mini Kit (Qiagen, 74104). RNA-Seq library generation and sequencing was outsourced to the London Regional Genomics Centre at the University of Western Ontario. RNA-Seq FASTq files were processed and trimmed with Trim Galore 0.6.4 and cutadapt 2.10 with Python 3.6.5. Read quality was evaluated with FastQC and aligned with STAR 2.7.3a and mm10 genome as reference using the Mayo Biocluster. The read counts and the differential gene expression were evaluated by FeatureCounts (Galaxy Version 1.6.4) and DESeq2 (Galaxy Version 2.11.40.6). Genes with a base mean ≥ 1, log_2_ fold change > 1 or < –1, and an FDR of ≤ 0.05 were considered significantly differentially expressed. Heatmaps were generated in RStudio software using pheatmap version 1.0.12 and volcano plots generated using EnhacedVolcano.

### Image analysis.

Nuclear volume and surface area quantifications were measured using the 3D objects counter feature in Fiji (ImageJ; NIH). The 8-bit images were taken and then the smooth feature was applied to normalize heterogenous DAPI staining. Next, nuclear volume and surface area were measured by applying 3D objects counter; nuclear threshold was applied manually to include maximum DAPI pixels while excluding background and detection with a minimum filter size of 50. Aggregated or partial nuclei were excluded. For cytoplasmic volume, total volume was taken in a similar fashion using ViaFluor 488 (Biotium, 70062), as a measure of cytoplasm volume (total volume – nuclear volume). For H&E tissue image analysis, slides were scanned by the Mayo Clinic Pathology Research Core with an Aperio ScanScope Slide Scanner. Mouse and TMA images were taken in SVS format and analyzed using QuPath (https://doi.org/10.1038/s41598-017-17204-5). Due to H&E staining heterogeneity, thresholding parameters were modified when appropriate. Thresholding was optimized to ignore background and detect nuclei while reducing nuclear fragmentation/merging. For whole-tissue imaging, eight 1,500 × 1,500 μm boxes were randomly placed on normal or cancer pancreata, preferentially avoiding regions containing stroma and islets. Nuclei were detected by optical density, threshold was applied, and images were analyzed. These same regions of interest (eight 1,500 × 1,500 μm boxes) were further analyzed using Delaunay cluster feature 2D measurement in QuPath to gain internuclear distance for each genotype. For TMAs, regions of normal or cancer pancreas were selected from cores, nuclei were detected by hematoxylin stain, threshold was applied, and regions were analyzed. For nuclear size analysis of human tissue, total control cells analyzed included 56,046 cells from 5 cases; for KRAS WT PDAC, there were 89,336 cells from 5 cases; and for KRAS^G12D^ PDAC, 79,679 cells were quantified from 5 cases. PanIN areas were identified on H&E images from KC mice (*n* = 11). Due to the lower prevalence of PanIN areas, there is reduced nuclei count compared with tumor KC mice — *n* = 27,680 versus *n* = 500,001 nuclei, respectively. QuPath analysis was used as described earlier for CSA analysis.

### Cell culture, transfections, and treatments.

Standard incubation conditions of 37°C and 5% CO_2_ were used in all experiments. PANC-1 (catalog CRL-1469; American Type Culture Collection) cells were obtained from ATCC and grown in DMEM (Corning, MT-10013CV) supplemented with 10% FBS (Corning, MT-35-010-CV). Doxycycline inducible shKRAS and shNT PANC-1 (iPANC-1) cells were cultured as previously described by Vivekanandhan et al. (37). 1012U and 4292F iKRAS cells were obtained from Marina Pasca di Magliano (University of Michigan). Mouse cell lines were derived from the genetic crosses described in this manuscript (KC, KPC, KPCE); for complete description of cell line establishment see Collins et al. (38). 1012U and 4292F cells were cultured in RPMI-1640 (Corning, 10-040-CV) with 10% FBS, and doxycycline hyclate (MilliporeSigma, D9891) was used at 1 μg/mL to induce mutant KRAS. HPNE-p16shRNA-*KRAS^G12V^*, HPNE-shRNA-control, and HPNE were a gift from Paul J. Chiao (University of Texas, Houston, Texas, USA) and were grown in 70% DMEM, 20% M3 base media, 500 μg/mL EGF, 0.45% glucose (1.1M)m and 10% FBS. BxPC3 were obtained from ATCC (catalog CRL-1687), cultured in RPMI-1640, and supplemented with 10% FBS. Cell line Hs766T were obtained from ATCC (catalog HTB-134) and MiaPaCa-2 (catalog CRL-1420), and both cell lines were grown in DMEM and supplemented with 10%FBS. siRNA transfections were performed with RNAiMAX (Invitrogen, 13778500) or DharmaFECT (Horizon Discovery, T-2001-03), and plasmid transfections were performed with Lipofectamine 2000 (Invitrogen, 11668019), per manufacturers suggestions. Briefly, for knockdowns and plasmid transfections, cells were initially seeded at a concentration required to obtain 70% confluency for the next day. Cells were transfected with diluted transfection complex in Opti-MEM (Thermo Fisher Scientific, 31985062) and incubated for 24 hours (if cells were required to be induced, doxycycline was added at this time to obtain 72 hours of total induction at time). Following incubation, transfected cells were then split for desired assays and harvested 48 hours later. siRNAs used in these experiments were Mouse EMD FlexiTube (Qiagen, SI00170506), Mouse EMD ON-TARGETplus SMARTPool siRNA (Horizon Discovery, L-040132-01-0005), Human EMD FlexiTube (Qiagen, SI00002296), Mouse Lamin B1 ON-TARGETpool SMARTPool siRNA (Horizon Discovery, L-049021-01-0005), Mouse Lamin A/C ON-TARGETplus SMARTPool siRNA (Horizon Discovery, L-040758-00-0005), and Human KRAS ON-TARGETplus siRNA (Horizon Discovery, L-005069-00-0005). Working concentration for siRNAs was 40 nM. Plasmid transfections for *cMYC* and *CYCLIN D1^T286A^* were performed using Lipofectamine 2000 as transfection reagent (1 μg/1.5 μL DNA/lipofectamine ratio). For the MEKi assay, 1012U cells were deinduced (–Dox) for 48 hours, induced (+Dox) for 48 hours, and then treated with either vehicle (DMSO) or MEKi (10 μM) for 24 hours. MEKi concentration was used based on the acute duration of treatment to ensure effective pERK mitigation (39). Cells seeded on coverslips were fixed for IF, and cells collected for protein were lysed and processed as described previously for Western blot analysis.

### Tissue IF.

To assess endocrine, exocrine, and ductal pancreatic morphology of the mice, pancreata were harvested, fixed in 10% formalin, and embedded in paraffin. Sections were subsequently immunostained for insulin in β cells, glucagon in α cells, and α-amylase in acinar cells with Vectashield-DAPI mounting medium (Vector Laboratories, H-1000). Images were acquired at 5×–20× magnification by the Zeiss Axio Observer Z1 microscope (Carl Zeiss Microscopy, LLC), and percent β cell area was measured analyzed using ZenPro software (Carl Zeiss Microscopy, LLC). Refer to Supplemental Table 1 for antibody details.

### IHC and histopathology analysis.

Pancreas and spleen were collected from the mice and fixed in 10% neutral buffered formalin (Thermo Fisher Scientific, 427-098). Samples were processed by Mayo Clinic Histology Core Laboratory for paraffin embedding and sectioning for H&E, Feulgen, and pERK staining. For H&E staining, tissue slides were baked and run through xylene to remove paraffin. Tissue was then rehydrated and stained with hematoxylin and blued, after which they were counterstained with eosin. Tissues were then dehydrated, cleared, and mounted with mounting media. For Feulgen, hydrolysis was done with 1M HCl at 60°C for 10 minutes, before being immediately transferred to Schiff’s reagent for 30 minutes at room temperature. Counterstain was done using Fast Green FCF, and slides were dehydrated, cleared, and mounted. Slides for pERK were completed by Pathology Research Core (Mayo Clinic) using the Leica Bond RX stainer (Leica). Briefly, slides were rehydrated, and antigen retrieval was performed using BOND Epitope Retrieval Solution 1 for 20 minutes. Primary antibody pERK 1/2 or EMD (Supplemental Table 1) was incubated for 15 minutes. The Polymer Refine Detection System (Leica), which includes hydrogen peroxidase block, polymer reagent, 3,3′-diaminobenzidine (DAB), and hematoxylin was used for IHC visualization. Once complete slides were dehydrated in increasing concentrations of ethyl alcohol and cleared in xylene prior to permanent mounting. H&E tissue samples were blindly evaluated to determine incidence of tumor and grade of severity by pathologists. For human EMD IHC analysis of low-grade PDAC, 47,963 nuclei were quantified from 22 cases and, for high-grade PDAC, 50,056 nuclei were quantified from 15 cases.

### Statistics.

Assays were checked for normality and lognormality. When comparing 2 groups, a parametric unpaired 2-tailed *t* test was performed for datasets that passed normality, while for those that did not pass normality, a nonparametric 2-tailed Mann-Whitney *U* test was performed to determine statistical significance. Datasets with 3 groups were checked for normality, and if they passed, 1-way ANOVA was performed followed by Tukey’s multiple-comparison test to find statistical significance. If the data did not pass normality for datasets containing ≥ 3 groups, then a Kruskal-Wallis test was conducted and a Dunn’s test to determine statistical significance. Statistical analyses and corresponding graphical representation were done using GraphPad Prism 9 software. All data are composed of at least 3 independent experiments. *P* values less than 0.05 were considered statistically significant.

### Study approval.

Mice were housed in pathogenic-free conditions and maintained in facilities approved by the American Association for Accreditation of Laboratory Animal Care in accordance with current regulations and standards of the United States Department of Agriculture, Department of Health and Human Services, and NIH/IACUC. All the procedures for mouse experiments were performed in accordance with protocols approved by the IACUC of Mayo Clinic (protocol no. A00003299).

### Data availability.

Data used in the figures and supplemental figures are available in the Supporting Data Values file. Raw data from RNA-Seq (BioProject ID PRJNA1112965) analyses have been deposited in the NCBI Sequence Read Archive data repository.

## Author contributions

Conceptualization was contributed by LFF, DLM, KLW, and MEFZ. Methodology was contributed by LFF, DLM, EJT, ANS, and MEFZ. Investigation was contributed by LFF, REV, ANS, KCLN, EJT, DRP, BRT, BC, MDT, LLA, RMC, RPG, CEH, MRB, and CLP. Visualization was contributed by LFF, DLM, DWD, CLP, AVM, KJR, and MEFZ. Funding acquisition was contributed by MEFZ. Project administration was contributed by MEFZ. Supervision was contributed by MEFZ. Writing original draft was contributed by LFF and MEFZ. Review and editing were contributed by all authors.

## Supplementary Material

Supplemental data

Unedited blot and gel images

Supporting data values

## Version 1 06/10/2025

In-Press Preview

## Version 2 07/22/2025

Electronic publication
