# Supplementary material for: Emerin is an effector of oncogenic KRAS-driven nuclear dynamics in pancreatic cancer
Source: JCI Insight. 2025 Jun 10;10(14):e187799. doi: 10.1172/jci.insight.187799 (PMC12288966; doi:10.1172/jci.insight.187799)
Supplement: Supplemental data [file jciinsight-10-187799-s243.pdf]

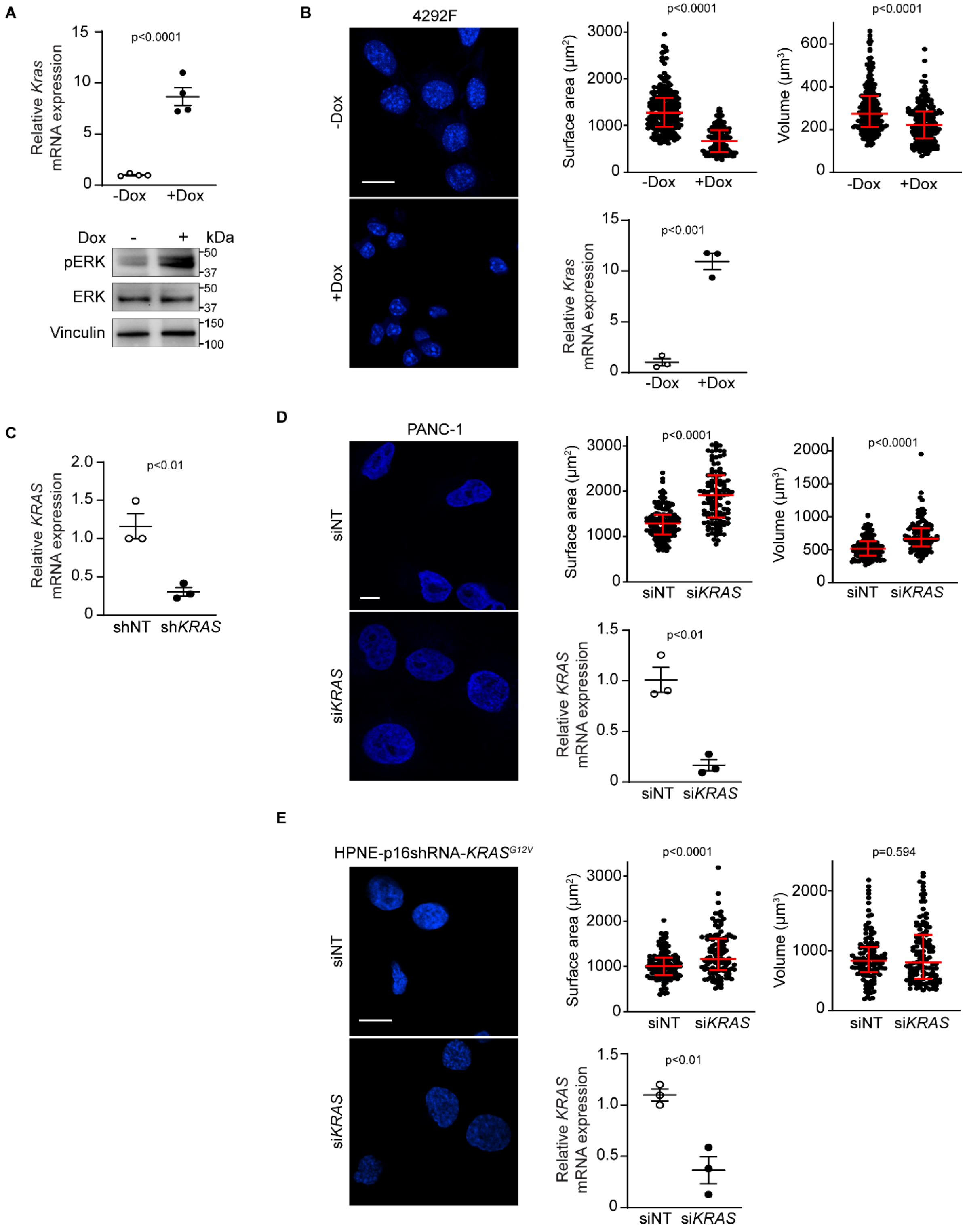

Supplemental Figure 1. **Mutant KRAS^{G12D} induces nuclear size reduction in human and**

mouse PDAC lines. A) *Kras* gene expression quantification by qRT-PCR in -/+ Dox 1012U cells relative to *mPrt*/*Tbp* and normalized to control group (n=4/group). Western blot of pERK and total ERK levels with Vinculin loading control. Scatter dot plot: mean with SEM. **B)** Confocal images of DAPI stained -/+ Dox 4292F cells. Scale bar, 20µm. Quantitation of nuclear size (Dox n=211; +Dox n=177 nuclei). Scatter dot plot: median with interquartile range. Quantification of *Kras* gene expression relative to *mPrt*/*Tbp* by qRT-PCR (n=3). Scatter dot plot: mean with SEM. **C)** *KRAS* gene expression quantification by qRT-PCR in iPANC-1 cells harboring Dox-inducible KRAS-shRNA and non-targeted control (shNT) (n=3). Scatter dot plot: mean with SEM. **D)** DAPI stained PANC-1 cells treated with either siNT or siKRAS with nuclear surface and volume quantification (siNT n=143; siKRAS n=115 nuclei). Scale bar, 10µm. Scatter dot plot: median with interquartile range. *KRAS* gene expression quantification by qRT-PCR in PANC-1 cells transfected siNT or siKRAS relative to *HPRT/TBP* (n=3). Scatter dot plot: mean with SEM. **E)** Confocal images of DAPI staining HPNE-p16shRNA-$KRAS^{G12V}$ scale bar, 20µm with qRT-PCR of relative *KRAS* gene expression (n=3) and quantification nuclear size (siNT n=118; siKRAS n=115 nuclei). **A-E)** Significant difference was determined by Mann-Whitney test (surface area and volume) and Student's t test (mRNA expression).

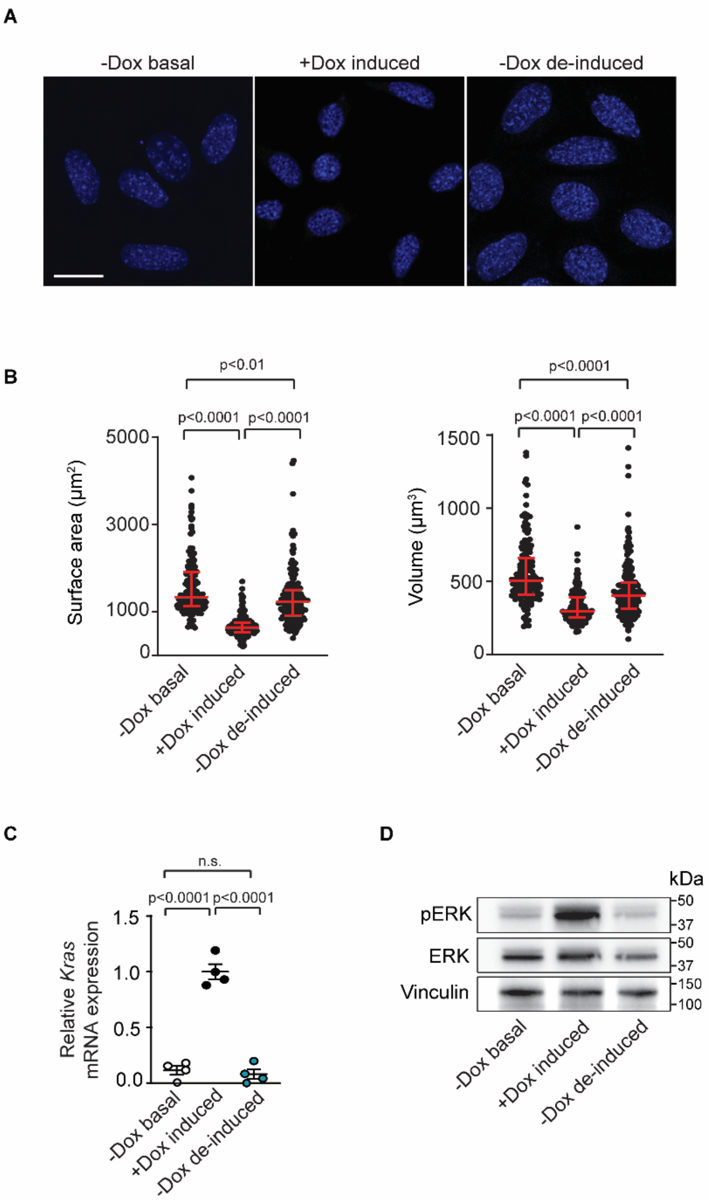

Supplemental Figure 2. **KRAS-induced nuclear size reduction in PDAC cells is reversible.** **A)** Confocal of DAPI staining in 1012U cells in basal, KRAS-induced (+Dox), and de-induced (-Dox) conditions. Scale bar, 20µm. **B)** Quantification of nuclear surface area and volume in basal, induced, and de-induced 1012U cells (basal=150; induced=203; de-induced=183 nuclei). Scatter dot plot: median with interquartile range. Significant difference was determined by Kruskal-Wallis followed by Dunn's multiple comparison test. **C)** *Kras* gene expression quantification by qRT-PCR in basal, induced, and de-induced 1012U cells (n=4). Scatter dot plot: mean with SEM. Significant difference was determined by ANOVA followed by Tukey's multiple comparison test. **D**) Western blot of pERK, total ERK and loading control of Vinculin in basal, induced, and de-induced 1012U cells.

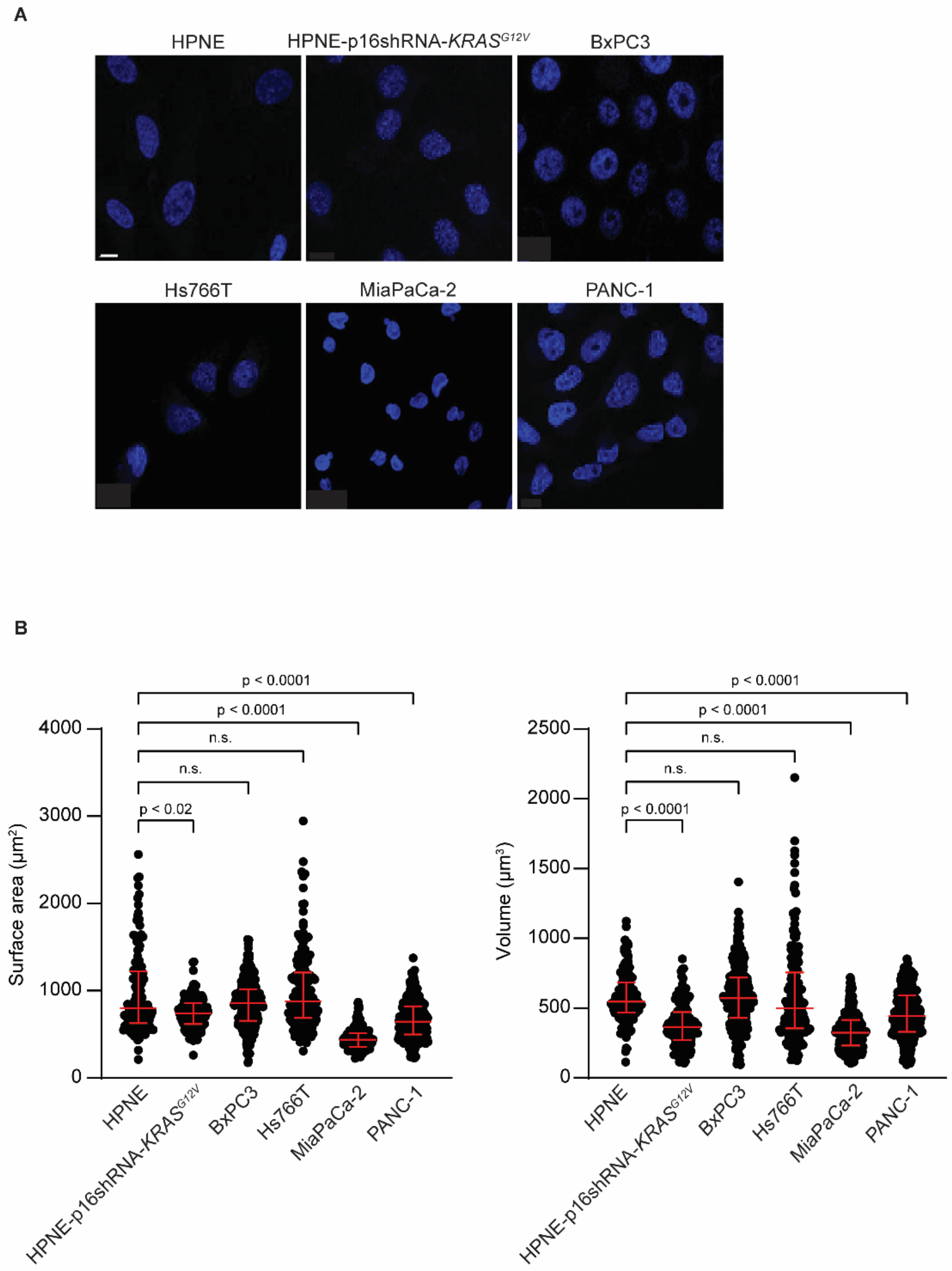

Supplemental Figure 3. **PDAC cell lines with various KRAS mutations show reduction in nuclear size. A)** Representative IF images (DAPI stained) of PDAC cell lines that are normal (HPNE), wild type for KRAS (BxPC3 and Hs766T), or contain KRAS mutations G12V, G12C, or G12D (HPNE-p16shRNA-*KRAS*^{G12V}, MiaPaCa-2, PANC-1, respectively). Scale bar, 10µm. **B)** Quantification of nuclear surface area and volume of PDAC cell lines. (Nuclei totals: HPNE n=146, HPNE-p16shRNA-KRAS^{G12V} n=164, BxPC3 n=295, Hs766T n=173, MiaPaCa-2 n=247, PANC-1 n=287). Scatter dot plot: median with interquartile range. Significance was determined using Kruskal-Wallis test followed by Dunn's multiple comparison test.

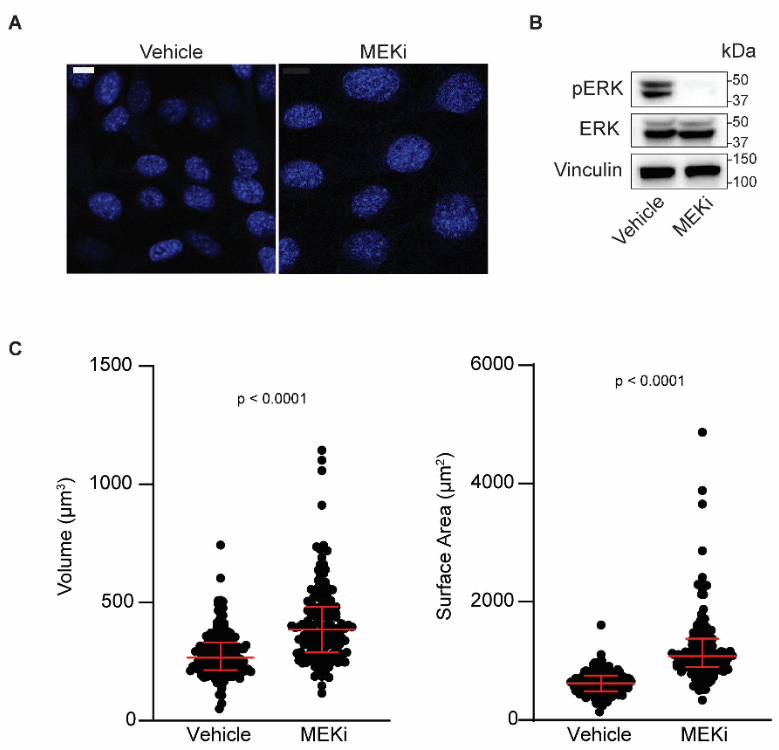

Supplemental Figure 4. **Inhibiting MEK/ERK signaling cascade prevented decrease of nuclear size. A)** Confocal image of nuclear DAPI stain of 1012U cells in +Dox condition treated with vehicle (DMSO) or MEKi (10uM, U0126). Scale bar, 10µm. **B)** Western blot of 1012U cells under +Dox condition treated with vehicle (DMSO) or MEKi probing for pERK, total ERK, and Vinculin as a loading control. **C)** Nuclear volume and surface area quantification (vehicle n=153; MEKi n=154 nuclei). Scatter dot plot: median with interquartile range. Significance was determined using Mann-Whitney test.

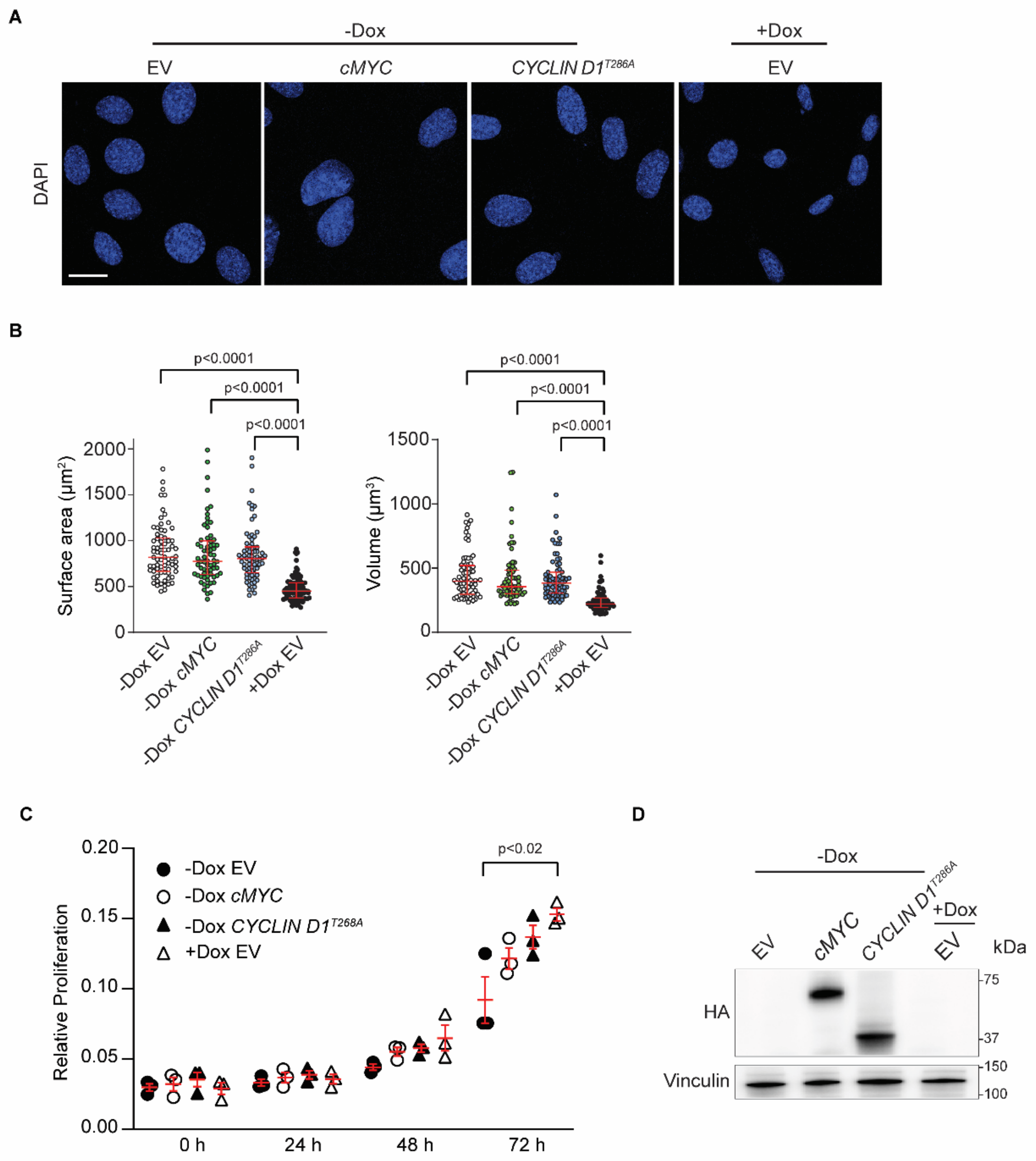

Supplemental Figure 5. Nuclear size reduction is specific to mutant KRAS signaling activation. A) Confocal microscopy of DAPI staining in -Dox 1012U cells transfected with empty vector (EV), HA-tagged *cMYC* or *CYCLIN D1*^{T286A} compared with +Dox 1012U cells transfected with EV. Scale bar, 20µm. **B)** Quantification of nuclear surface area and volume for conditions in panel A (-Dox (EV)=72; *CYCLIN D1*^{T286A}=74; *cMYC*=66; +Dox (EV)=82 nuclei). Scatter dot plot: median with interquartile range. Significance was determined using Kruskal-Wallis test followed by Dunn's multiple comparison test. **C)** -Dox 1012U cells transfected with empty vector (EV), HA-tagged *cMYC* or *CYCLIN D1*^{T286A} compared with +Dox 1012U cells transfected with EV were collected for Cyquant cell proliferation measurement at 0, 24, 48, and 72h post transfection or Dox treatment (n=3/timepoint). Interleaved scatter plot: mean with SEM. Significant difference was determined by one-way ANOVA followed by Tukey's multiple

comparison test. **D)** Western blot of HA-tag with vinculin loading control in 1012U -/+ Dox condition that were transfected with HA-tagged empty vector (EV), *cMYC*, or *CYCLIN D1*^{T286A}.

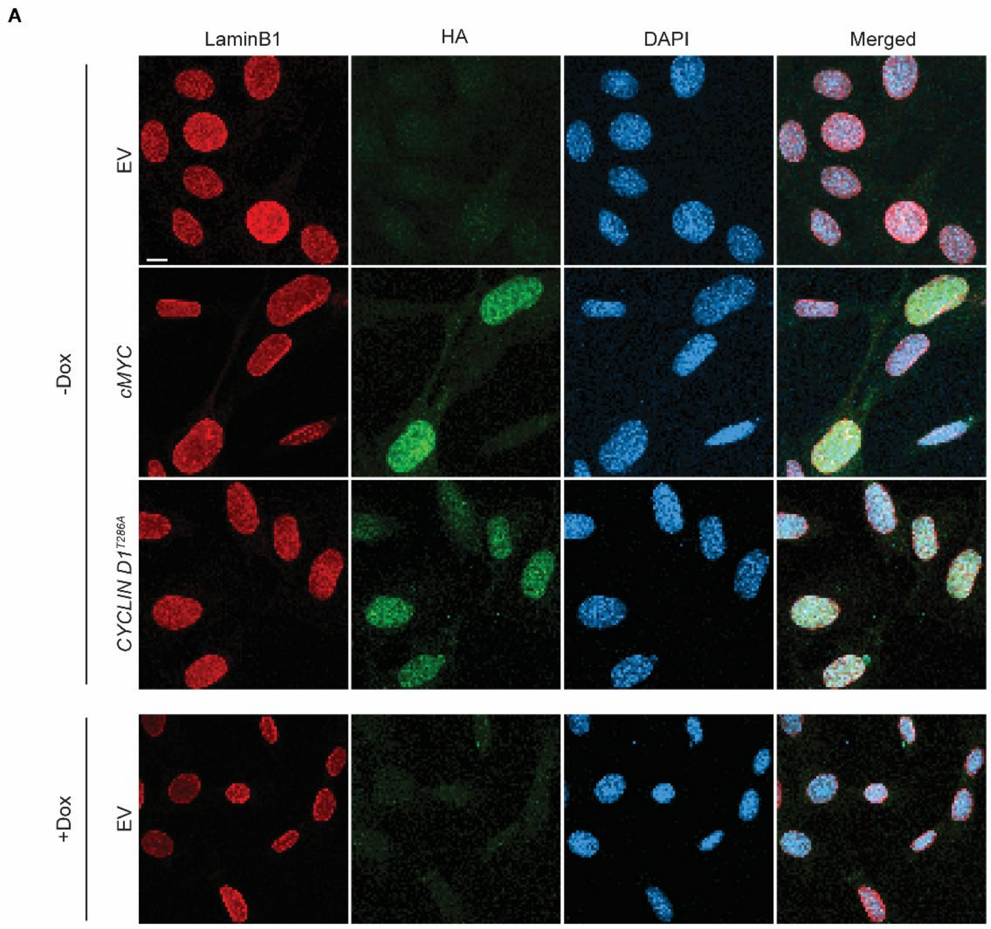

Supplemental Figure 6. Nuclear localization of *cMYC* and mutant *CYCLIN D1* in KRAS inducible cell lines. A) IF images of 1012U -/+Dox transfected with plasmids, landmark protein LaminB1 (red), localization of overexpression of EV, *cMYC*, or *CYCLIN D1*^{T286A} HA-tag (green), DAPI (blue) and merged. Scale bar, 10 µm.

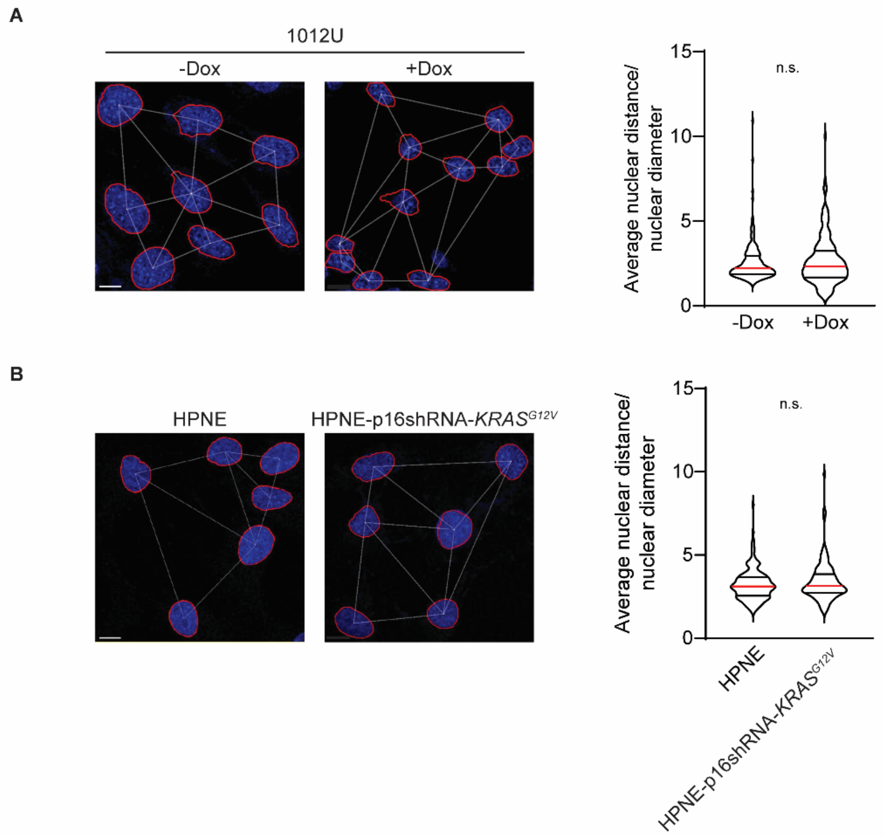

Supplemental Figure 7. **PDAC cell line Delaunay cluster relative to nuclear diameter. A)** DAPI staining of 1012U cell line (-/+Dox condition) nuclear recognition (red) and Delaunay measurements (white lines) with quantitative (-Dox=192; +Dox=212 nuclei) distance relative to nuclear diameter. Violin plot: median with interquartile range. Significance was determined using Mann-Whitney test. **B)** DAPI staining of HPNE and HPNE-p16shRNA-*KRAS*^{G12V} cell lines with nuclear recognition (red) and Delaunay measurements (white lines) with quantitative (HPNE=172; HPNE- *KRAS*^{G12V} =140 nuclei) distance relative to nuclear diameter. Violin plot: median with interquartile range. Significance was determined using Mann-Whitney test.

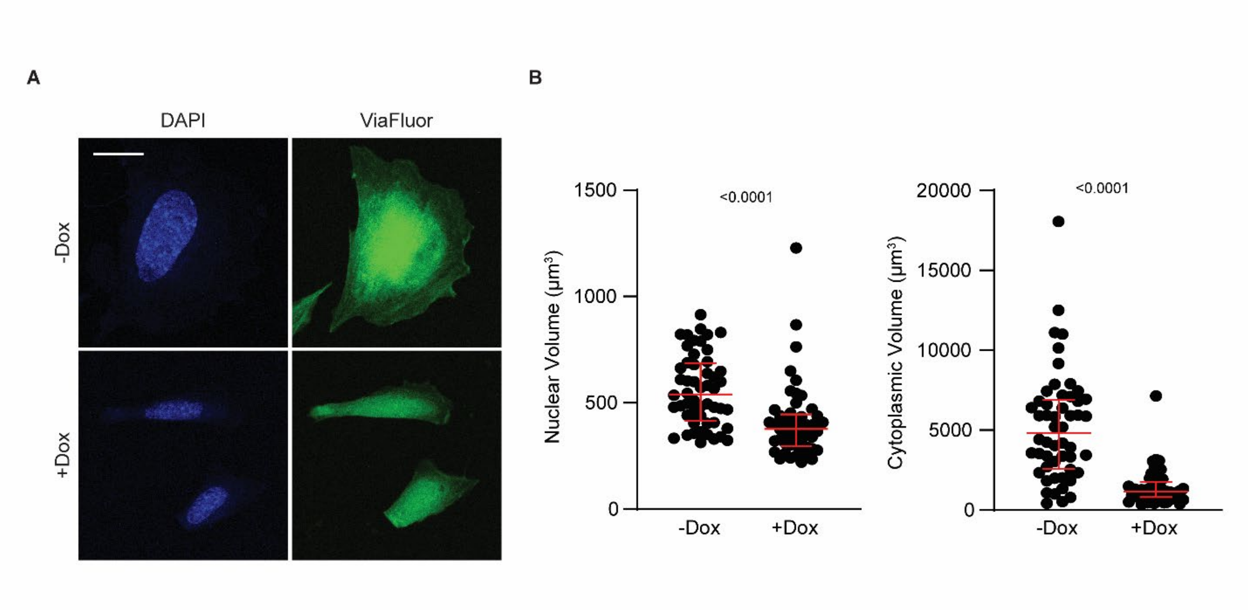

Supplemental Figure 8. **Total cell volume significantly decreases in KRAS-induced cells. A)** Confocal representative images of 1012U -/+ Dox condition. Cells stained with DAPI (blue) and ViaFluor (green) for nuclear to cytoplasmic quantification. Scale bar, 20 µm. **B)** Quantitative measurements of nuclear volume and cytoplasmic volume in -/+ Dox conditions (n=3) (-Dox=52; +Dox=49 nuclei). Scatter dot plot: median with interquartile range. Statistical significance was determined by Mann-Whitney test.

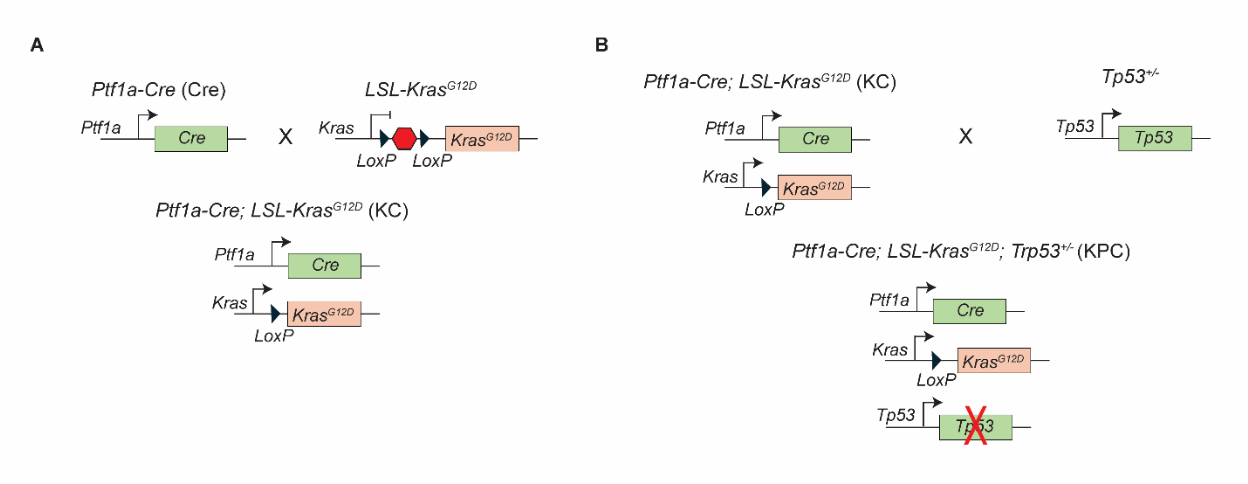

Supplemental Figure 9. Schematics of the KC and KPC crosses. A) Schematic representing animal crosses to generate model *Ptf1a-Cre; LSL-Kras*^{G12D} (KC) mice using *Ptf1a-Cre* (Cre) with *LSL-Kras*^{G12D}. **B)** Schematic representing animal crosses to generate model *Ptf1a-Cre; LSL-Kras*^{G12D}; *Trp53*$^{+/-}$ (KPC) mice using *Ptf1a-Cre; LSL-Kras*^{G12D} (KC) with *Trp53*$^{+/-}$ mice.

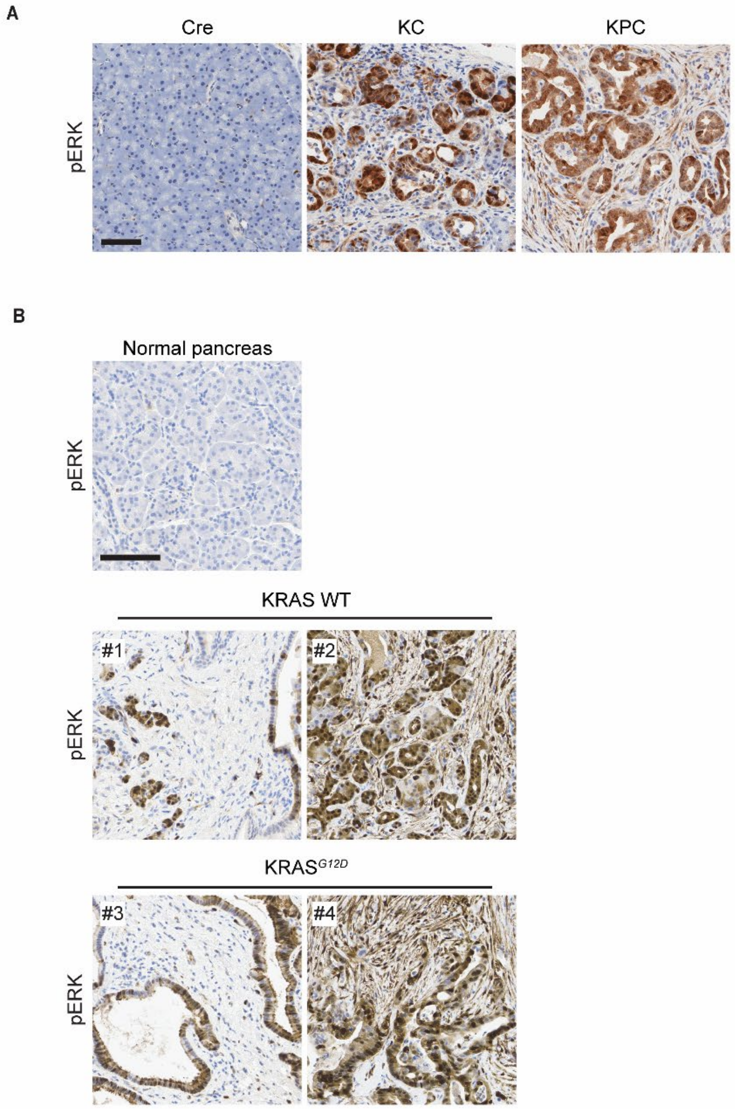

Supplemental Figure 10. Immunohistochemistry of pERK in mouse and human PDAC. A) IHC of mouse pancreas tissue stained for pERK for Cre, KC, and KPC models. Scale bar, 100 µm. **B)** IHC of human pancreas tissue probed for pERK. Normal pancreas tissue, KRAS wild type (n=5), and KRAS^{G12D} (n=5). Case #1 and #2 are both wild type (WT) for KRAS with case #2 containing a ALK chromosomal rearrangement which can contribute to elevated pERK. Case #3 and #4 are both contain KRAS^{G12D} and represent the range of pERK expression.

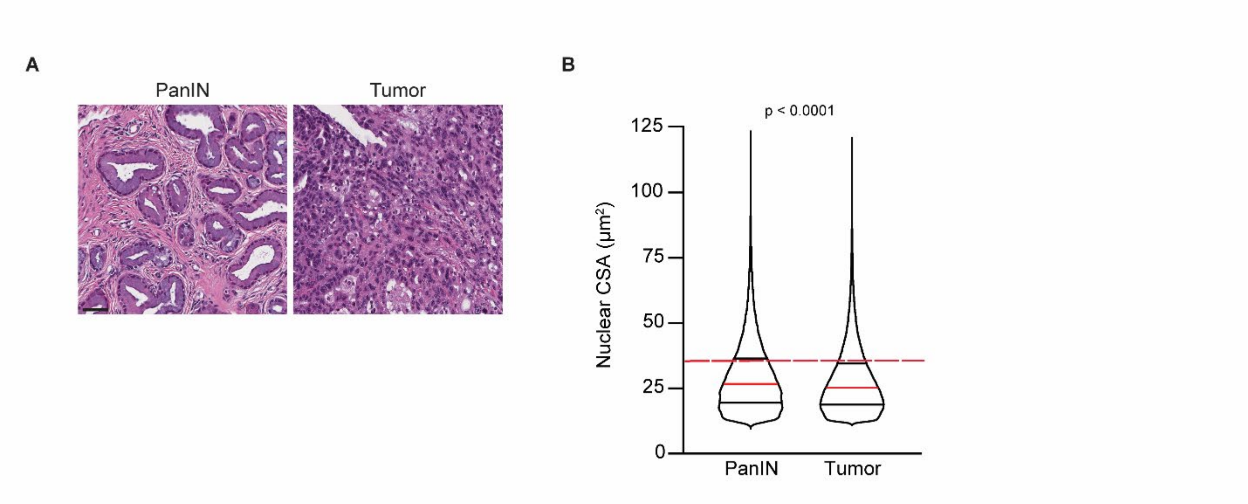

Supplemental Figure 11. PanIN's nuclear size is greater compared to tumor nuclei. A) H&E representative images of KC mice (n=11) with PanIN or tumor. Scale bar, 60µm. **B)** Quantification of CSA (PanIN n=27,680; tumor n=500,001 nuclei). Red dotted line indicates median CSA of Cre nuclei for reference. Violin plot: median with interquartile range. Significant difference was determined by Mann-Whitney test.

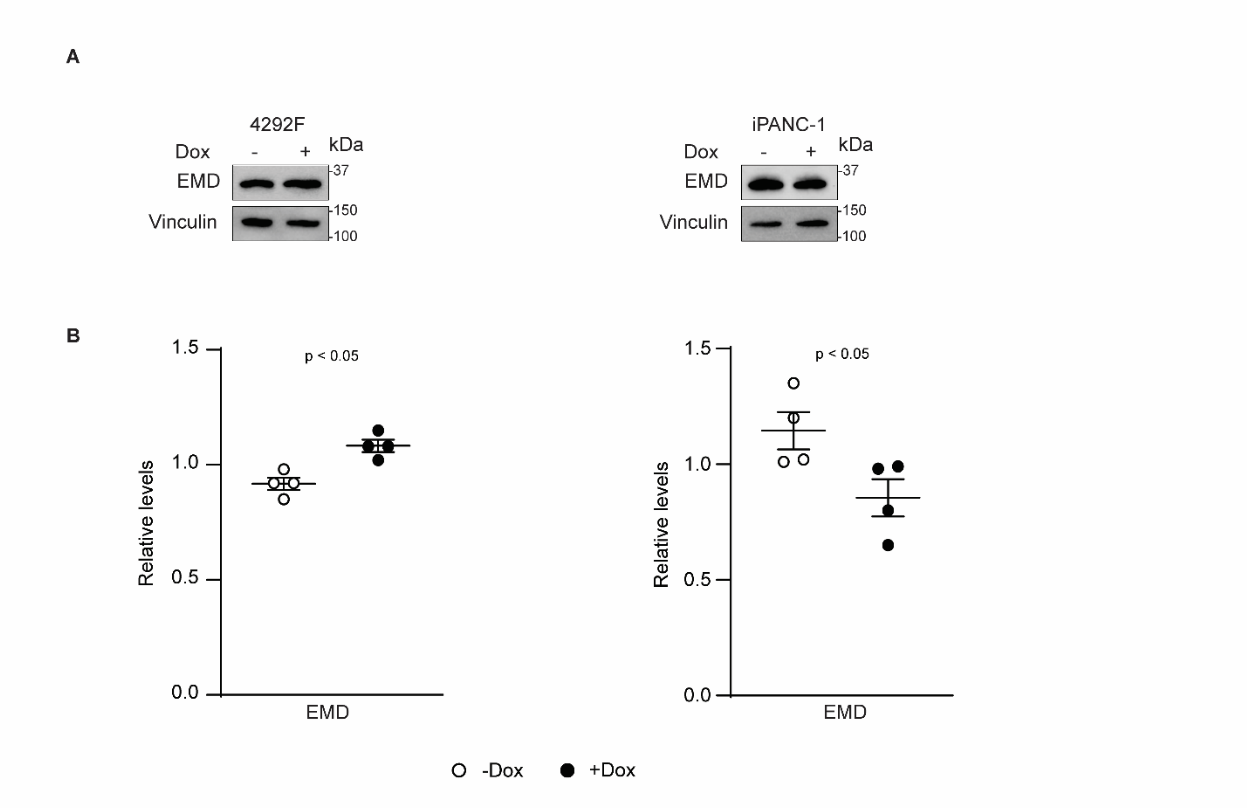

Supplementary Figure 12. Mutant KRAS is required to control EMD protein levels. A) Western blot of EMD protein with Vinculin loading control in mouse cell line 4292F and human cell line iPANC-1 in -/+Dox condition. **B)** Densitometry of Emerin protein levels normalized to Vinculin (n=4/cell line), 4292F and iPANC-1. Scatter dot plot: mean with SEM. Significance was determined by Mann-Whitney test.

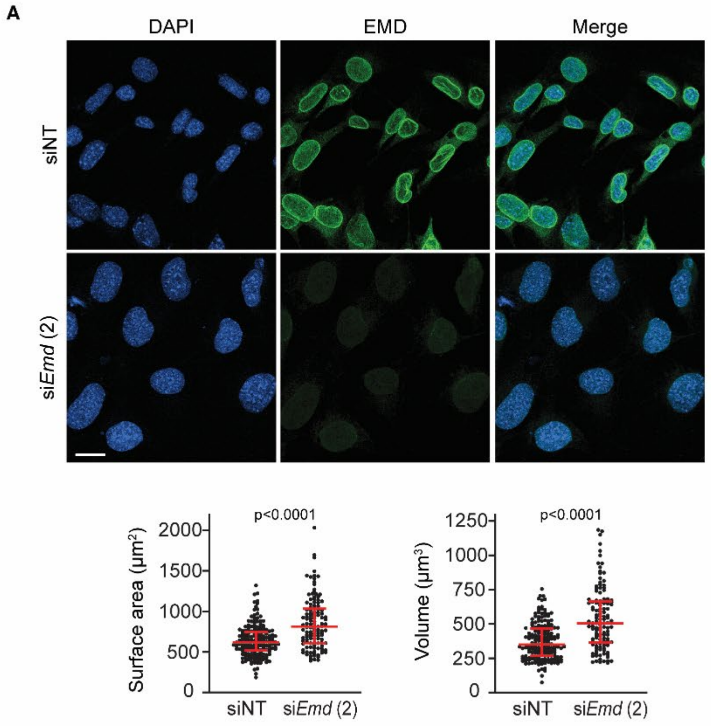

Supplemental Figure 13. Emerin depletion results in an increased nuclear size in KRAS mutant cells. A) IF of 1012U cells previously treated with doxycycline were transfected with an siRNA SMARTPool for *Emd* or control non-targeting siRNA (siNT). Scale bar, 10µm. Quantification of nuclear surface area and volume (siNT=187; si*Emd*=124 nuclei). Scatter dot plot: median with interquartile range. Significant difference was determined by Mann-Whitney test.

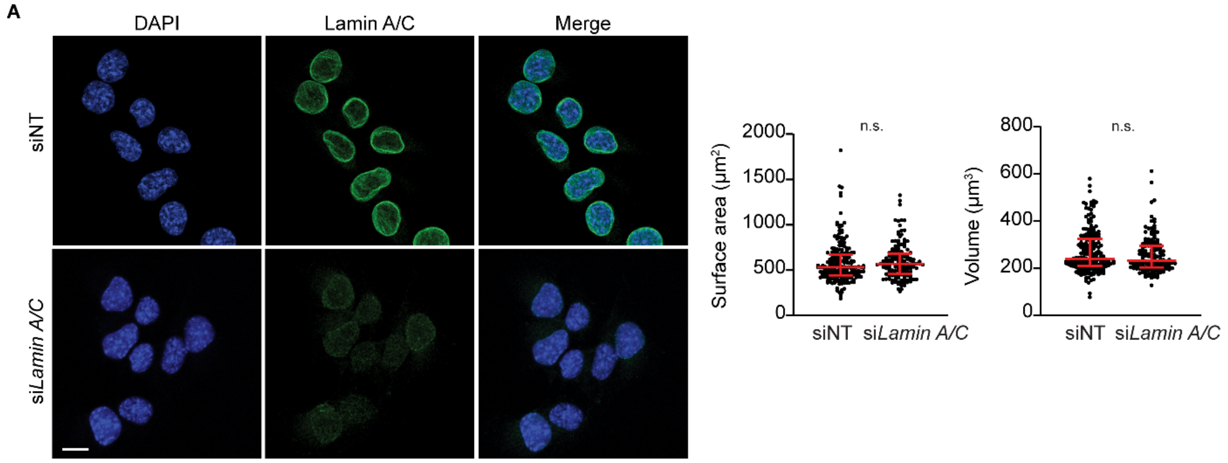

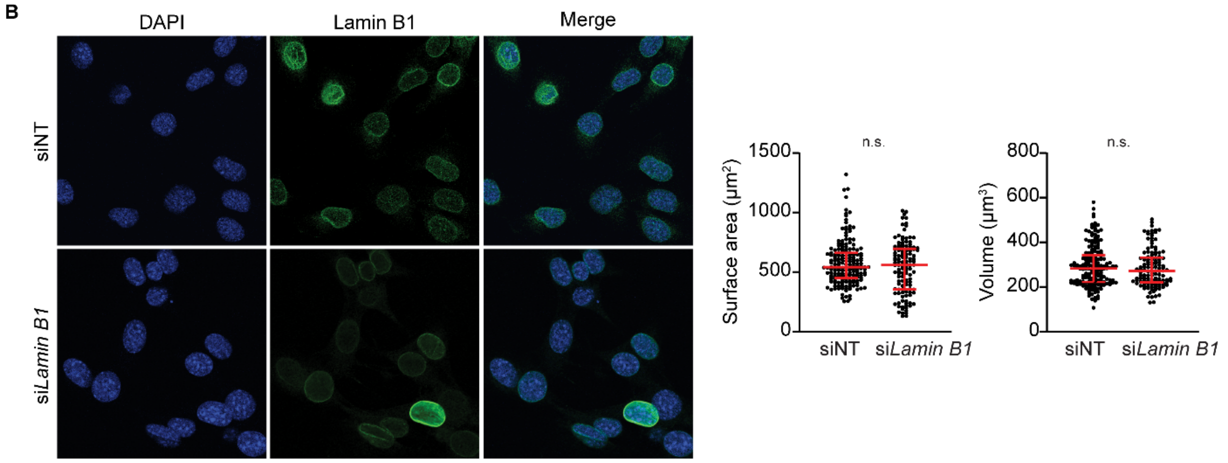

Supplemental Figure 14. Knockdowns of *Lamin A/C* or *Lamin B1* do not affect nuclear size downstream of KRAS. A) Confocal images and analysis of nuclear surface and volume quantification of 1012U cells in +Dox condition transfected with siNT and si*Lamin A/C* (siNT n=211; si*Lamin A/C* n=147 nuclei). **B)** IF of 1012U +Dox transfected with siNT and si*Lamin B1* and quantification of nuclear surface and volume (siNT n=180; si*Lamin B1* n=125 nuclei). **A and B)** Scale bar, 10µm. Scatter dot plot: median with interquartile range. Significant difference was determined by Mann Whitney test.

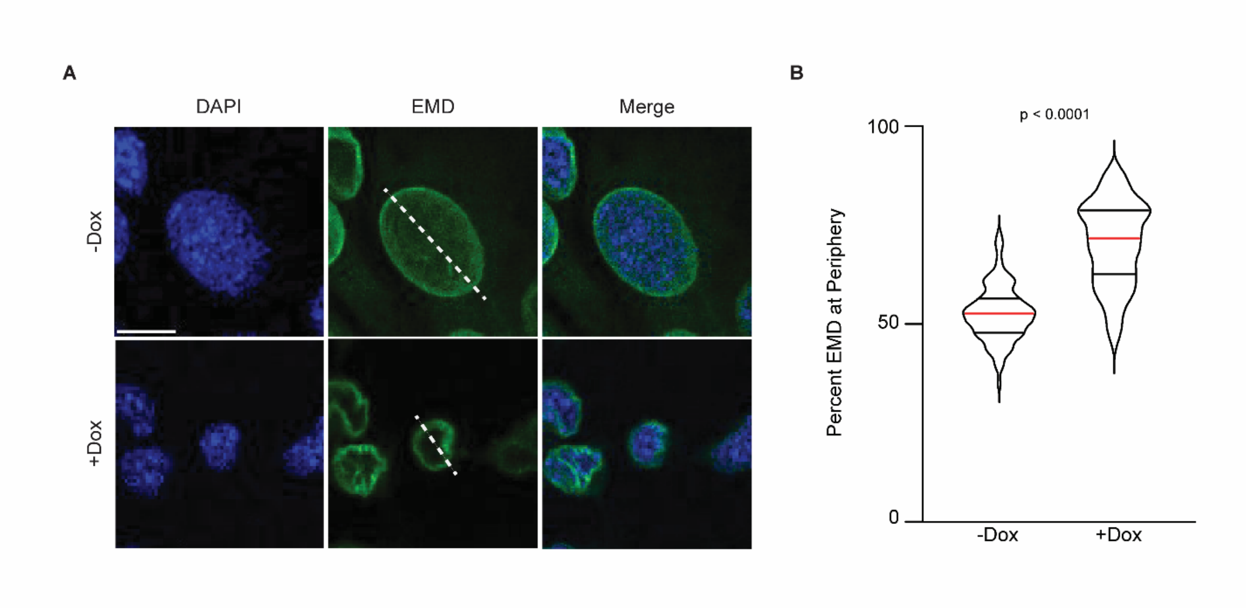

Supplemental Figure 15. KRAS induced Emerin localization to the nuclear periphery. A) IF of 1012U in -Dox and +Dox condition with EMD (Green) and nuclear DAPI (blue) staining. White dotted line indicates line of measurement through nucleus. Scale bar, 10µm. **B)** Line scan quantification (n=3) of peripheral EMD intensity (3 µm from nuclear edge) over total signal across the line (-Dox n=111; +Dox n=146 nuclei). Violin plot: median with interquartile range. Significant difference was determined by Mann-Whitney test.

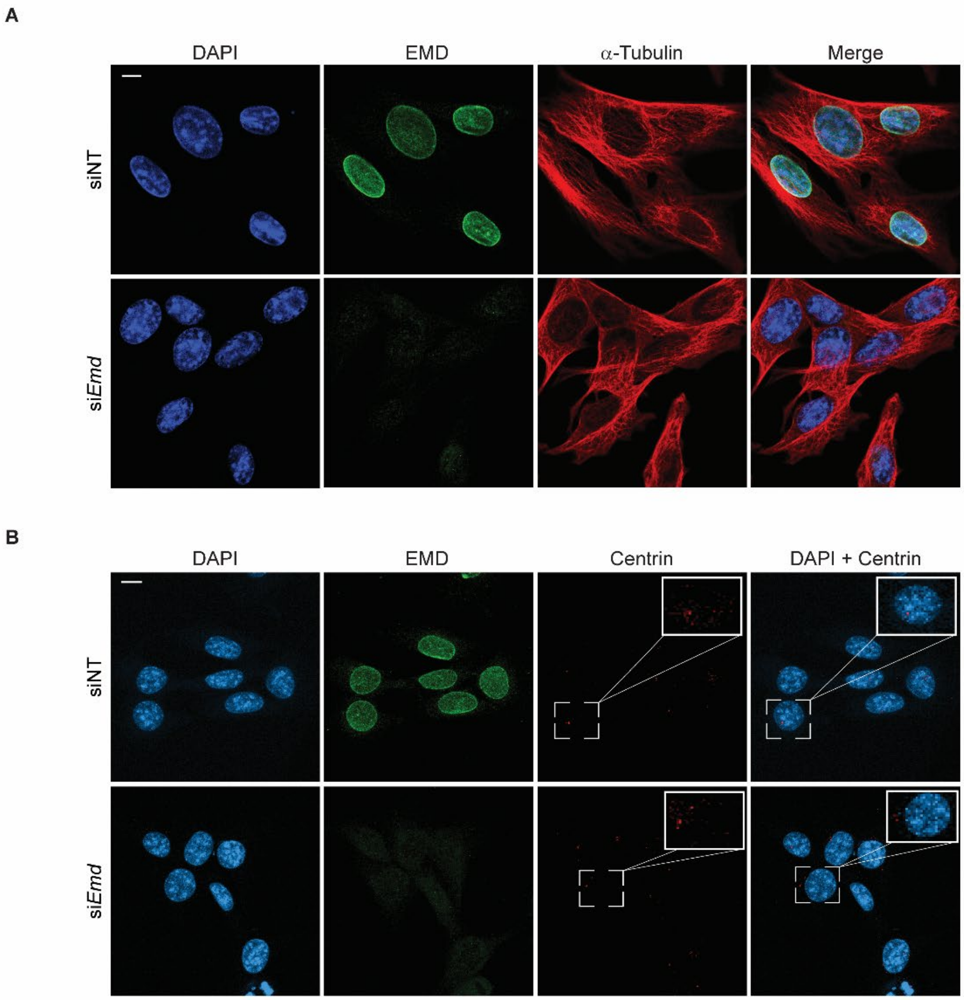

Supplemental Figure 16. Effect of knockdown of EMD on cytoskeleton and centrin organization. A and B) 1012U cells in +Dox condition treated with either siNT or si*Emd* with scale bar at 10μm. **A)** Representative confocal images DAPI (blue), EMD (green) and α-Tubulin (red). **B)** Representative images of confocal images nuclear DAPI (blue), EMD (green) and centrin (red). Solid white box shows zoomed in image of nucleus positive with centrin from dashed white box.

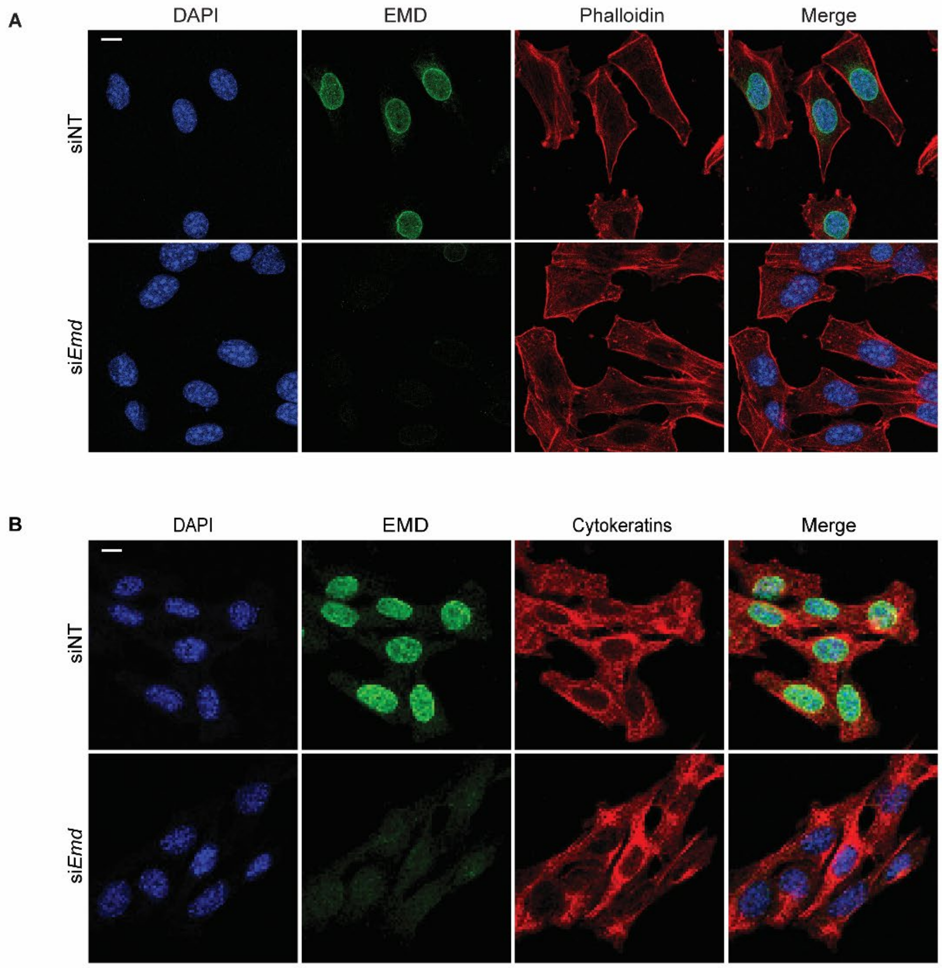

Supplemental Figure 17. Emerin knockdown does not affect actin filaments and cytokeratin organization. A and B) 1012U cells in +Dox condition treated with either siNT or siEmd with scale bar at 10µm. **A)** Representative confocal images with DAPI (blue), EMD (green) and Phalloidin (red) that indicates actin filaments present. **B)** Representative images DAPI (blue), EMD (green) and Cytokeratin/OSCAR (red).

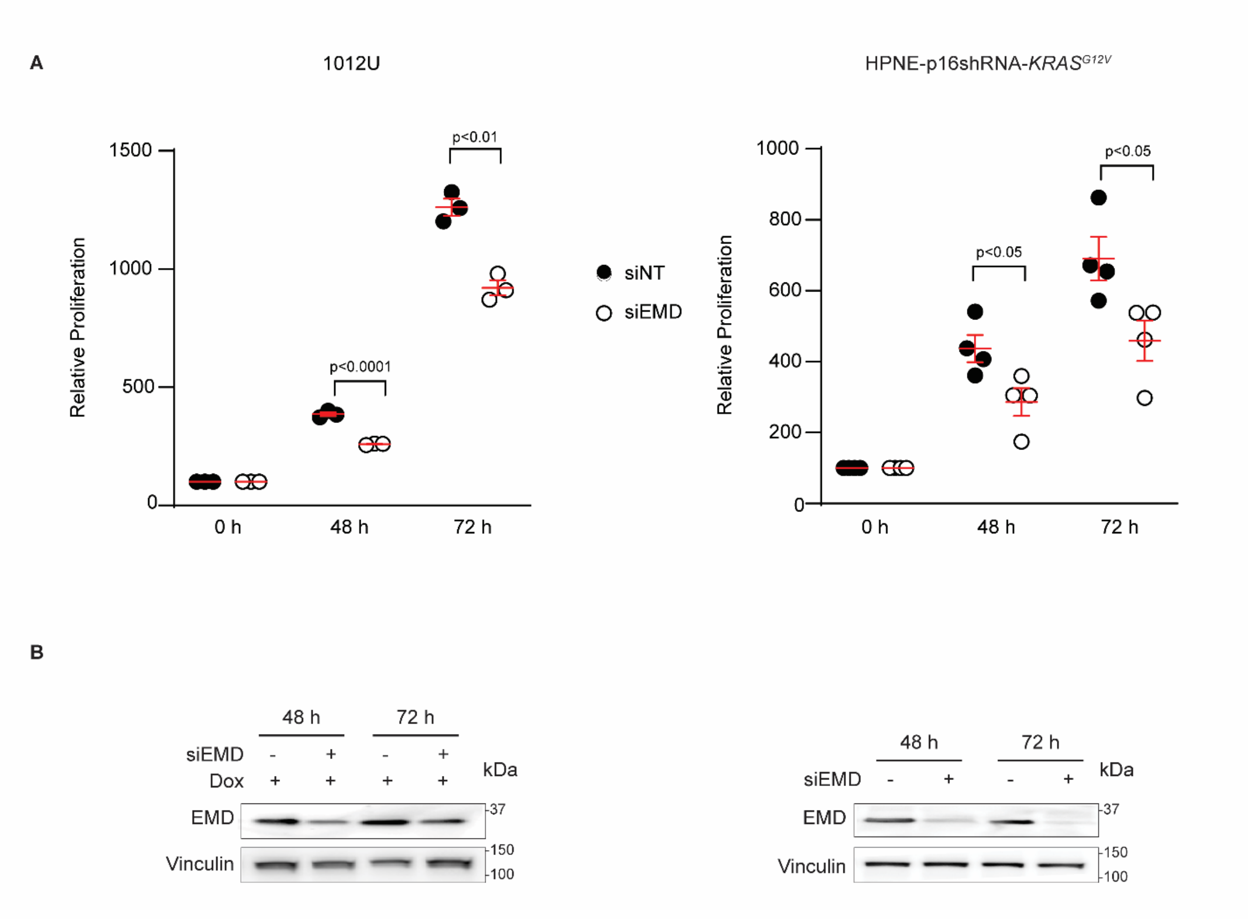

Supplemental Figure 18. Depletion of Emerin results in decreased proliferation. A) 1012U cells in +Dox condition treated with siNT or si*Emd* measured for Cyquant cell proliferation at 0, 48, and 72h (n=3/timepoint). HPNE-p16shRNA-$KRAS^{G12V}$ cells treated with siNT or si*Emd* measured for Cyquant cell proliferation at 0, 48, and 72h (n=4/timepoint). Dot plot: mean with SEM. Significance determined by Student t-test. **B)** Western blot of EMD and Vinculin at 48 and 72h for +Dox 1012U and HPNE-p16shRNA-$KRAS^{G12V}$.

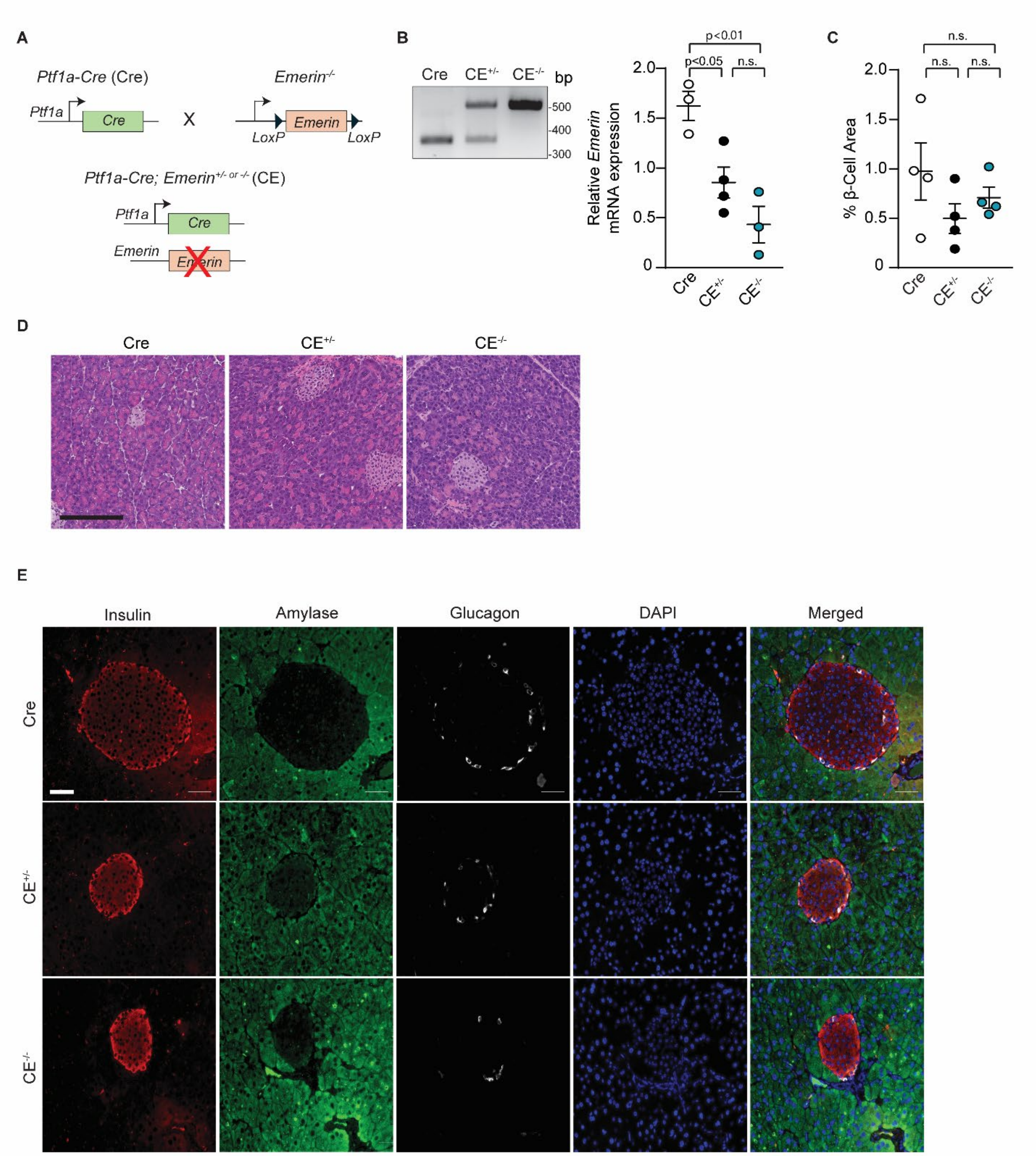

Supplemental Figure 19. Characterization of the pancreas-specific EMD conditional knockout model. A) Schematic representing animal crosses to generate model *Ptf1a-Cre; Emd*$^{+/-}$ (CE) mice using *Ptf1a-Cre* (Cre) with *Emd*$^{-/-}$ mice. **B)** PCR of mouse pancreas tissue to confirm proper recombination of *Emd* for mice models. Relative *Emd* gene expression in Cre, CE$^{+/-}$ and CE$^{-/-}$ mice models. Scatter dot plot: mean with SEM. Significance determined by ANOVA followed by Tukey's multiple comparison test. **C)** Percentage of ß-cell area present in Cre, CE$^{+/-}$ and CE$^{-/-}$ mice. Scatter dot plot: mean with SEM. Significance determined by ANOVA followed

by Tukey's multiple comparison test. **D)** Representative H&E stain of Cre, $CE^{+/-}$ and $CE^{-/-}$ mice. Scale bar, 200µm. **E)** IF images for insulin (ß-cells), amylase (exocrine pancreas), and glucagon (α-cells) and DAPI (nuclear) in pancreas tissue samples from Cre, $CE^{+/-}$ and $CE^{-/-}$ mice. Scale bar, 100µm.

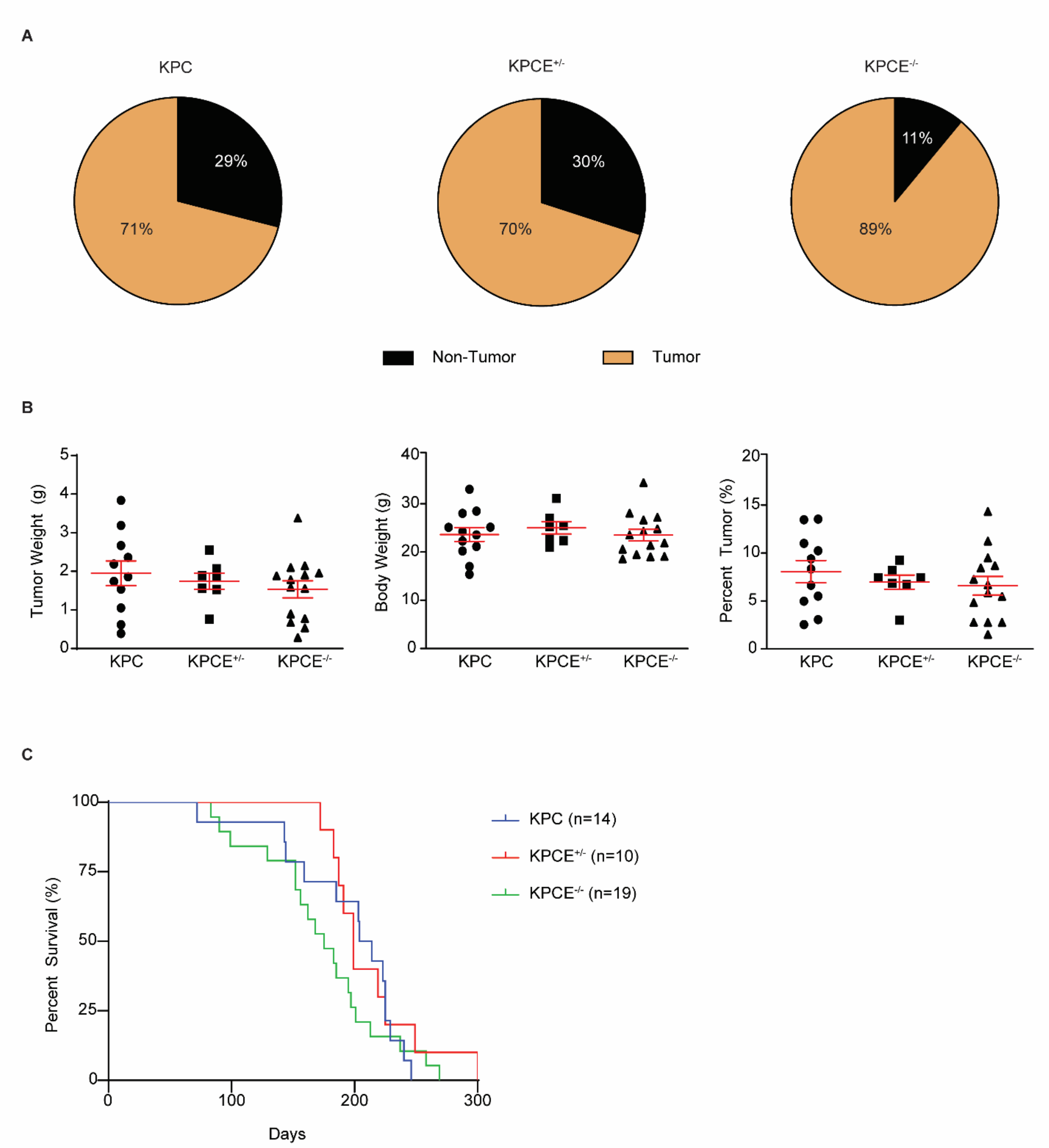

Supplemental Figure 20. Effect of EMD depletion on PDAC development. A) Percent tumor incidence of the mice KPC (n=11), KPCE$^{+/-}$ (n=7), KPCE$^{-/-}$ (n=14). **B)** Pancreas tumor weight (grams), body weight (grams) and ratio of tumor to body weight (percent) in KPC (n=11), KPCE$^{+/-}$ (n=7), KPCE$^{-/-}$ (n=14) mice. Dot plot: mean with SEM. Significant difference was determined by Kruskal-Wallis test. All graphs were not statistically different (p=0.64; 0.57; 0.60). **C)** Kaplan-Meier curve of KPC (n=14), KPCE$^{+/-}$ (n=10), KPCE$^{-/-}$ (n=19) mice. Log-rank Mantel-Cox test was used to determine significance (p=0.3028).

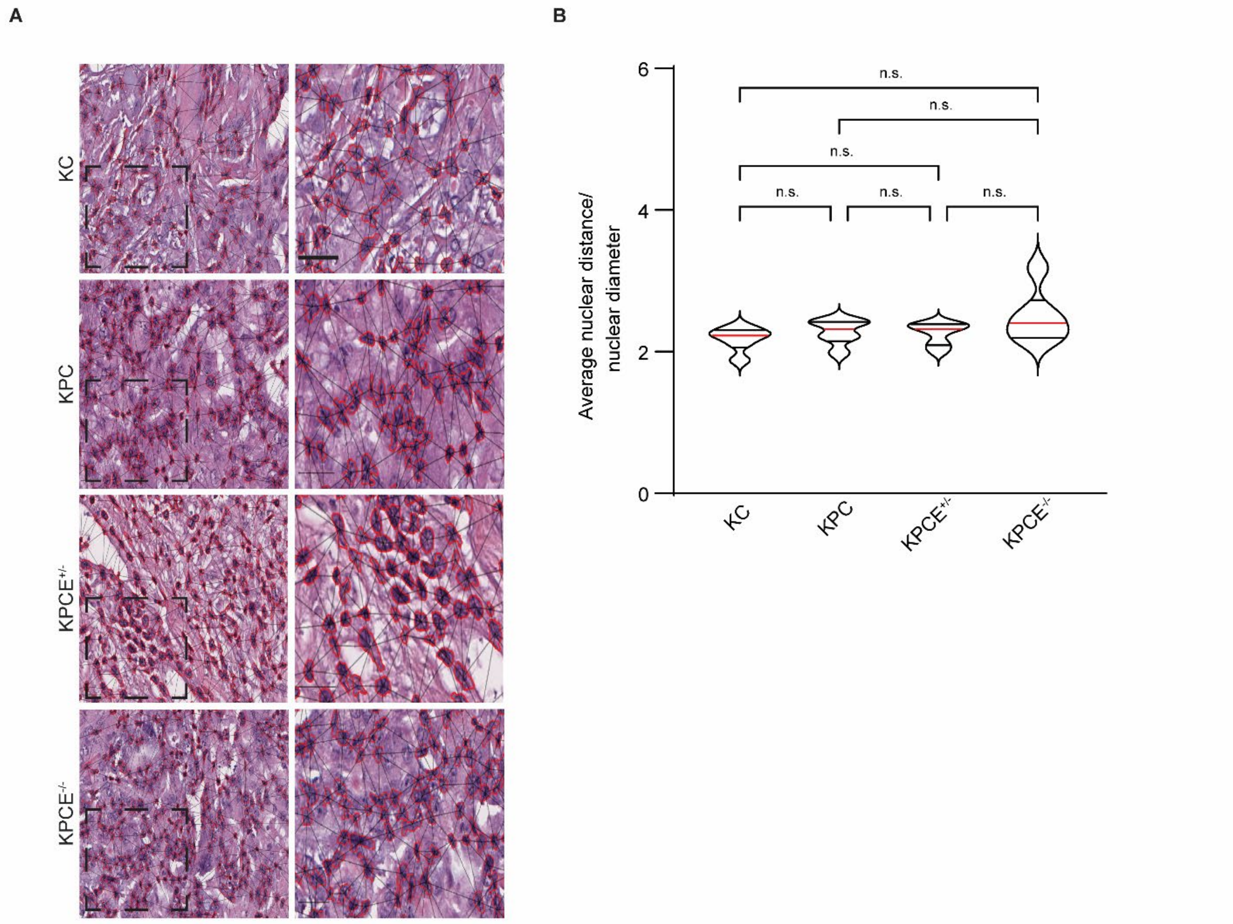

Supplemental Figure 21. In vivo ratios of inter-nuclear distance over nuclear diameter. A) Representative H&E images of each genotype indicating nuclei (red circle) and Delaunay cluster feature 2D measurement (black line). Left panel macroscopic view of cluster, right panel magnified area from black dashed box (in left panel) of inter-nuclear distance. Scale bar, 20µm. **B)** Quantitative crowding for each genotype. N=6 mice per group. Quantitative (KC=612,209; KPC=589,901; KPCE$^{+/-}$ n=572,026; KPCE$^{-/-}$ n=484,600 nuclei) distance relative to nuclear diameter. Violin plot: median with interquartile range. Significance was determined using Kruskal-Wallis test with Dunn's multiple comparisons test.

Target	Company	Catalog #	RRID#	Concentration
**Western Blot**				
Emerin	Abcam	ab156871		1:5000
Emerin	Proteintech	0351-1-AP	AB_2100056	1:5000
Lamin A+ Lamin C	Abcam	ab8984	AB_306913	1:2500
LAP2b	Proteintech	14651-1-AP	AB_2205440	1:1000
Lamin B1	Abcam	ab16048	AB_443298	1:7000
Lamin B2	Abcam	ab151735	AB_2827514	1:7000
Sun1	Aviva Systems Biology	ARP49929_P050	AB_1295001	1:1000
Sun2	Proteintech	27556-1-AP	AB_2880906	1:4000
Anti-HA	Roche	11867423001	AB_390918	1:1000
p44/42 (Erk 1/2)	Cell Signaling	9107S		1:1000
p44/42 MAPK (Erk1/2) (137F5)	Cell Signaling	4695S		1:1000
p-Erk/P-p44/42 MAPK (T202/Y204) (D13.14.4E) XP	Cell Signaling	4370S		1:1000
Vinculin	Bethyl	A302-535A	AB_1999080	1:10,000
Goat Anti-Rabbit IgG	EMD	AP132P	AB_90264	1:10,000
Goat Anti-Mouse IgG	EMD	AP124P	AB_90456	1:10,000
Goat Anti-Rat IgG	EMD	AP136P	AB_11214444	1:10,000
**Immunofluorescence**				
Emerin	Abcam	ab156871		1:150
Lamin A+ Lamin C	Abcam	ab8984	AB_306913	1:400
Lamin B1	Abcam	ab16048	AB_443298	1:250
Anti-HA	Cell Signaling	2367S		1:100
Insulin	Abcam	ab7842		1:100
Glucagon	Sigma-Aldrich	G2654		1:1000
α-Amylase	Sigma-Aldrich	A8273		1:100
Goat anti-Rabbit Alexa Fluor 594	Thermo Fisher Scientific	A-11037	AB_2534095	1:200
Goat anti-Rabbit Alexa Fluor 488	Molecular Probes	A-11008	AB_143165	1:200
Goat anti-Rabbit Alexa Fluor 568	Thermo Fisher Scientific	A-11011	AB_143157	1:200
Goat anti-Mouse Alexa Fluor 488	Thermo Fisher Scientific	A-11001	AB_2534069	1:200
Goat anti-Mouse Alexa Fluor 594	Thermo Fisher Scientific	A-11005	AB_2534073	1:200
Goat anti-Chicken Alexa Fluor 647	Thermo Fisher Scientific	A-21449	AB_2535866	1:200
**Immunohistochemistry**				
p44/42 MAPK (Erk1/2) (3A7)	Cell Signaling	9107S		1:1200
p-Erk/P-p44/42 MAPK (T202/Y204) (D13.14.4E) XP	Cell Signaling	4370S		1:1200

Supplemental Table 1: List of antibodies used for western blot, immunofluorescence and immunohistochemistry.
