# Supplementary material for: Emerin is an effector of oncogenic KRAS-driven nuclear dynamics in pancreatic cancer
Source: JCI Insight. 2025 Jun 10;10(14):e187799. doi: 10.1172/jci.insight.187799 (PMC12288966; doi:10.1172/jci.insight.187799)
Supplement: Unedited blot and gel images [file jciinsight-10-187799-s244.pdf]

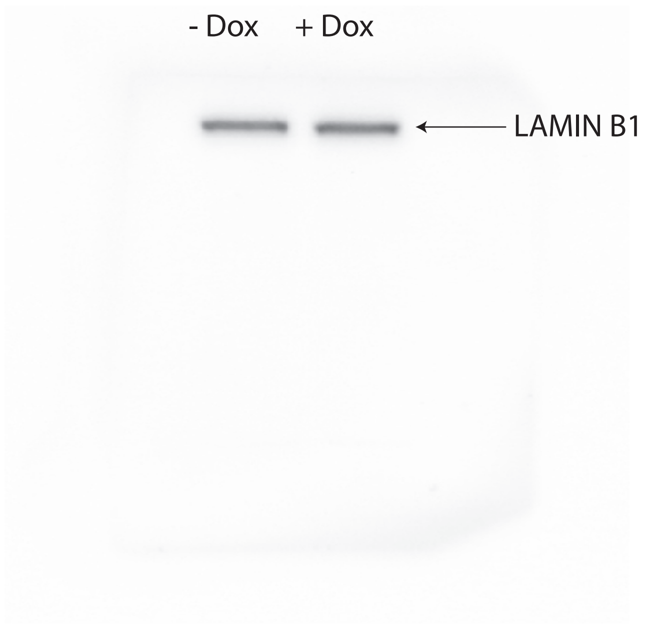
- Dox
+ Dox
LAMIN B1

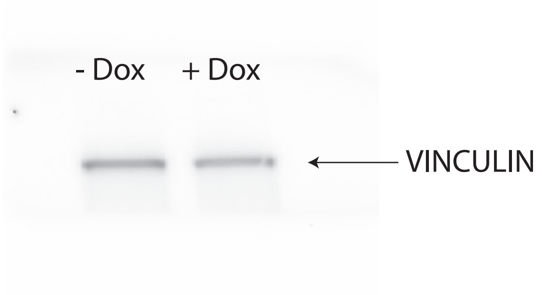
- Dox
+ Dox
VINCULIN

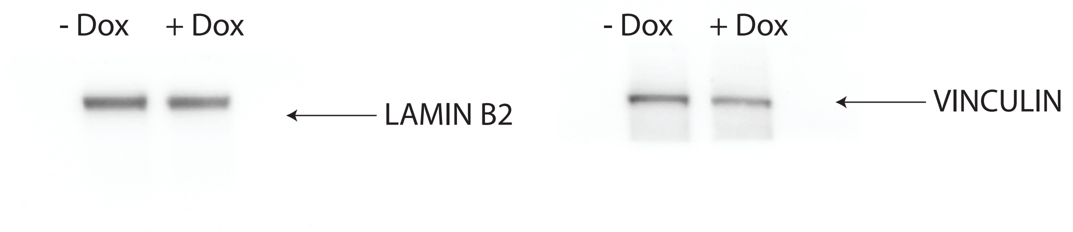
- Dox
+ Dox
LAMIN B2
- Dox
+ Dox
VINCULIN

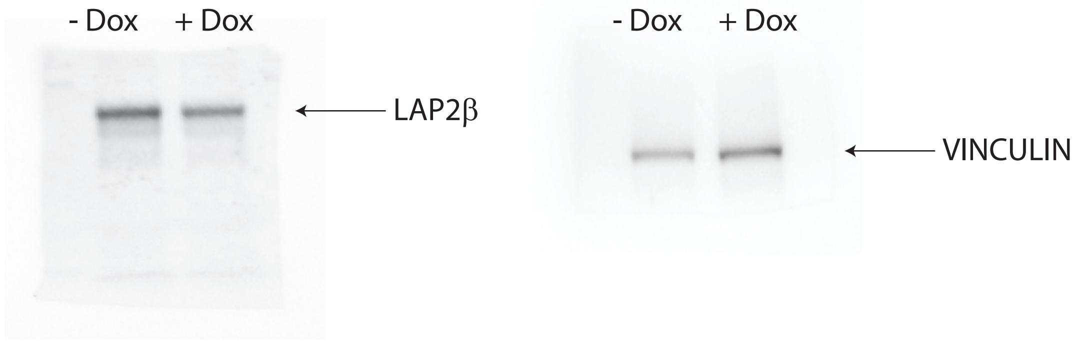
- Dox
+ Dox
LAP2β
- Dox
+ Dox
VINCULIN

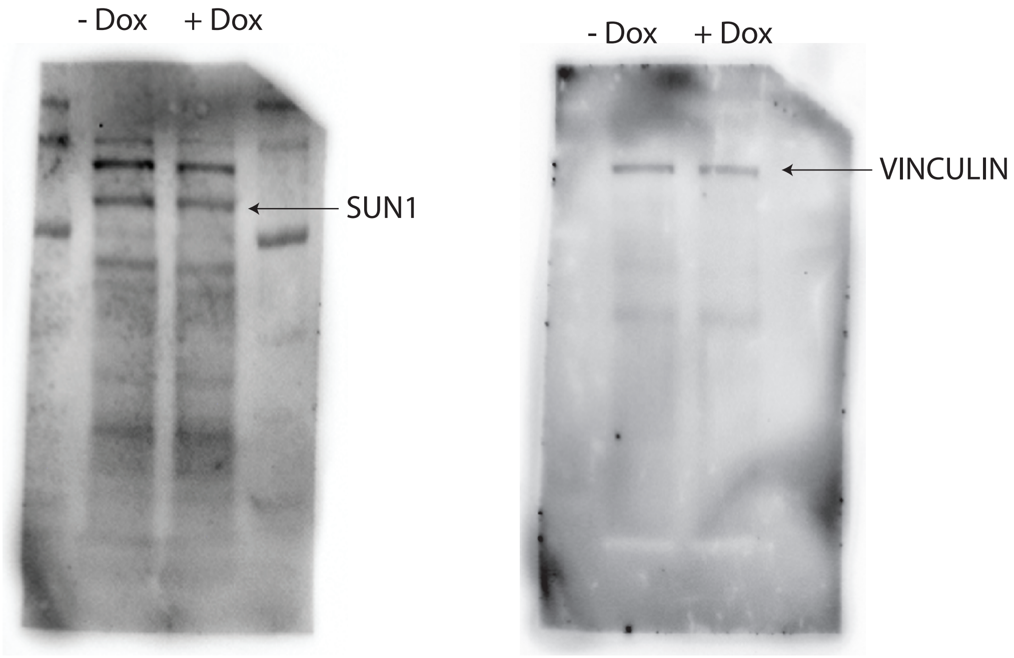
- Dox
+ Dox
SUN1
- Dox
+ Dox
VINCULIN

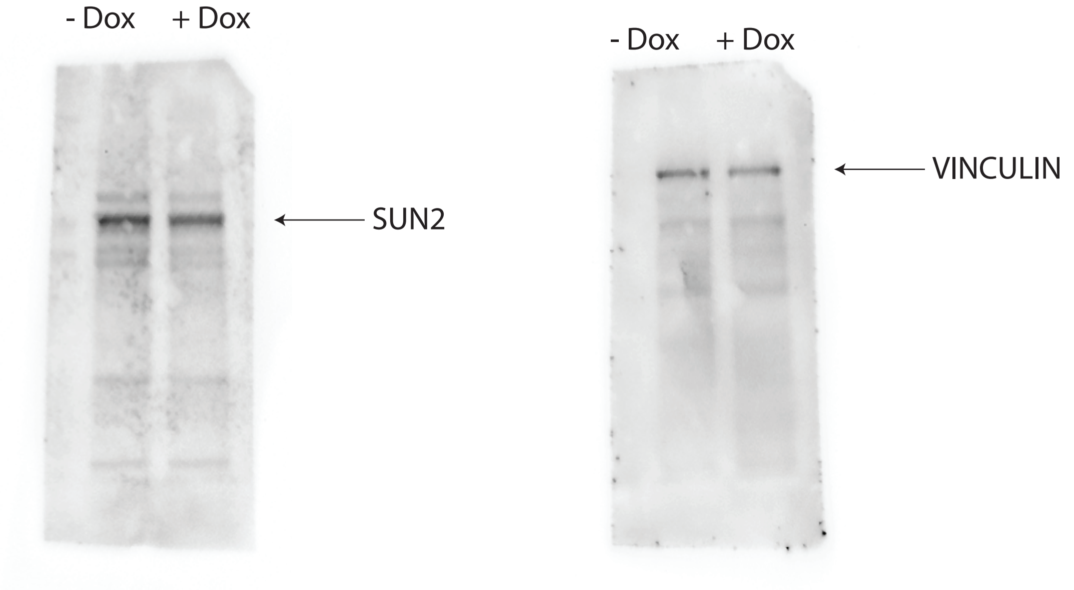
- Dox
+ Dox
SUN2
- Dox
+ Dox
VINCULIN

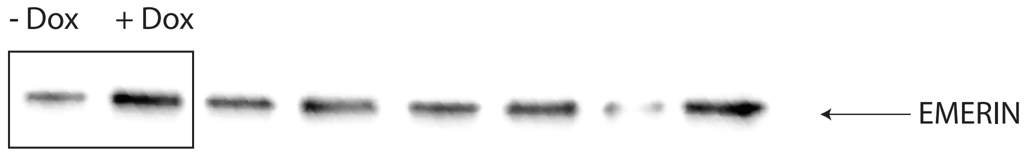
- Dox
+ Dox
EMERIN

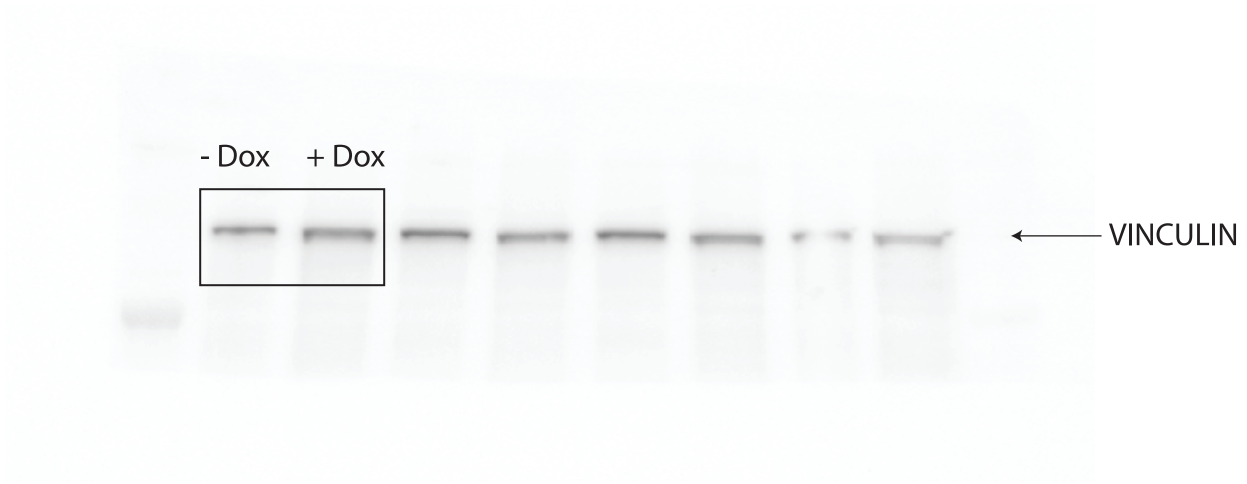
- Dox
+ Dox
VINCULIN

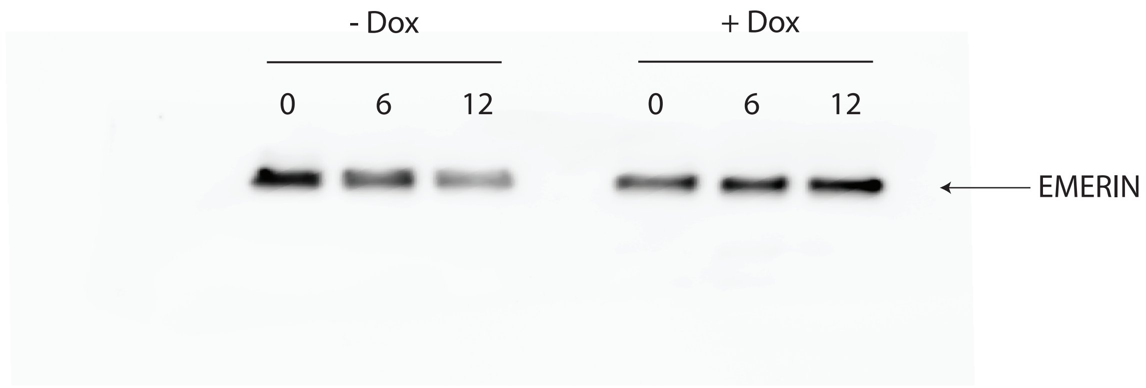
- Dox
+ Dox
0
6
12
0
6
12
EMERIN

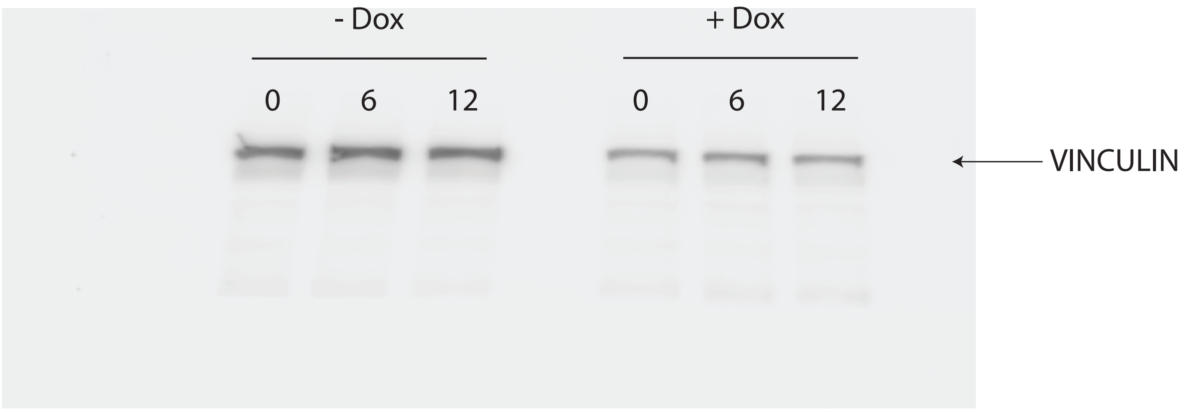
- Dox
+ Dox
0
6
12
0
6
12
VINCULIN

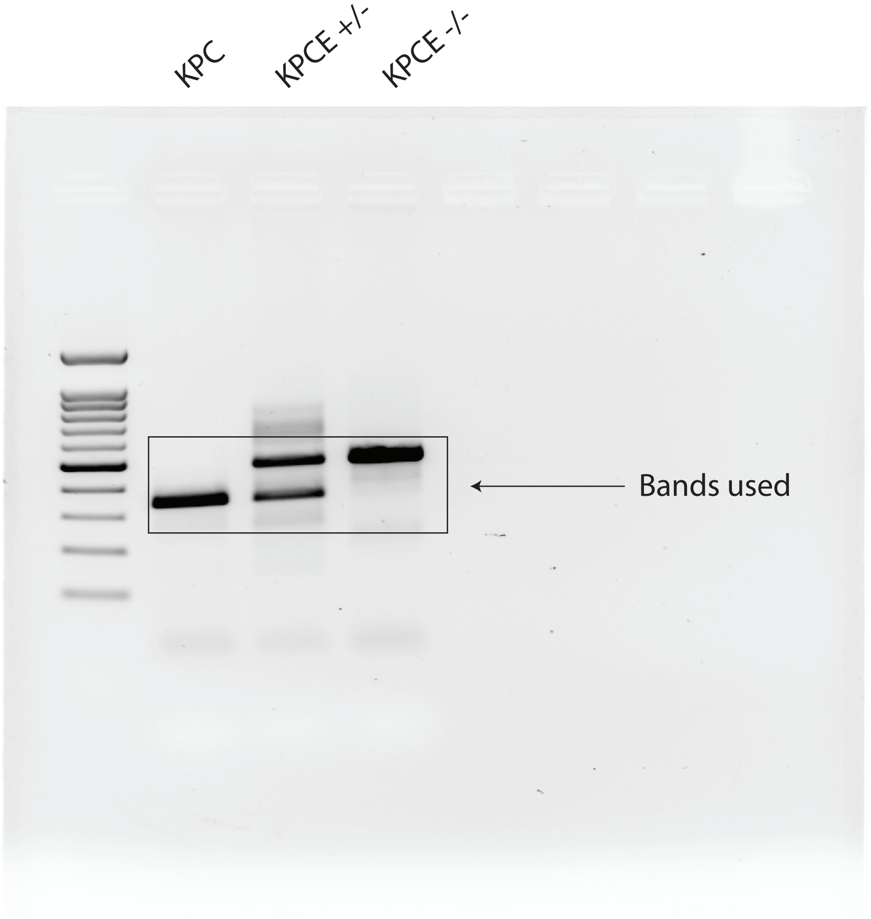
KPC
KPCE +/-
KPCE -/-
Bands used

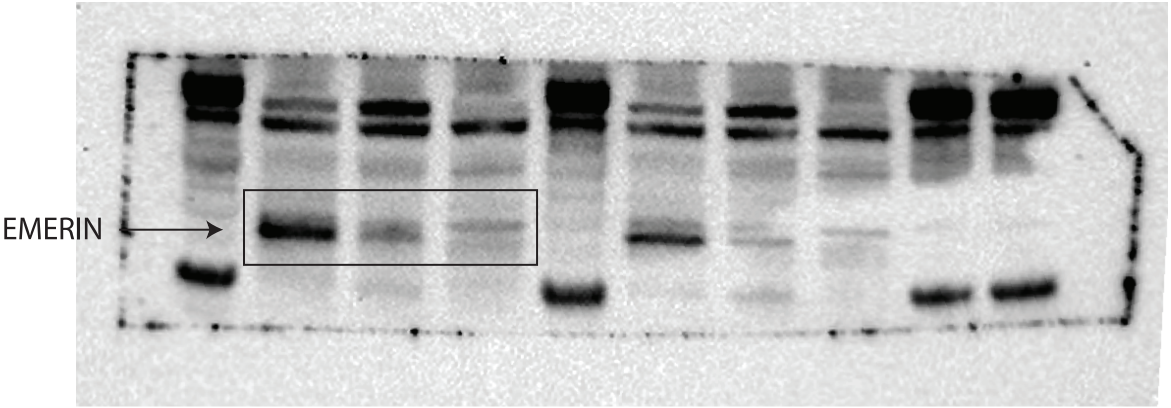
EMERIN

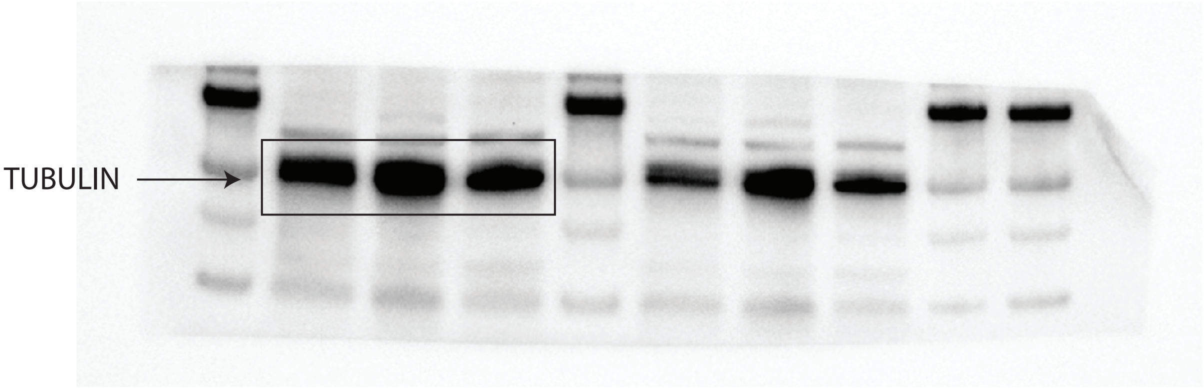
TUBULIN

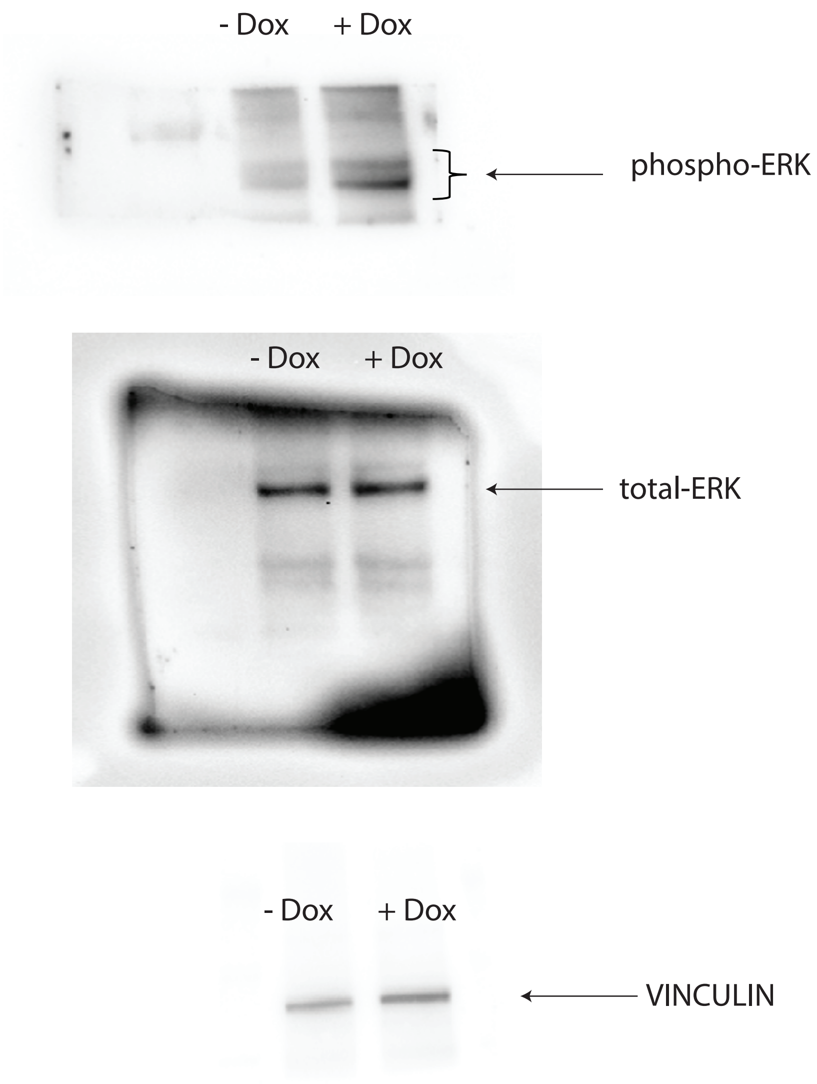
- Dox
+ Dox
phospho-ERK
- Dox
+ Dox
total-ERK
- Dox
+ Dox
VINCULIN

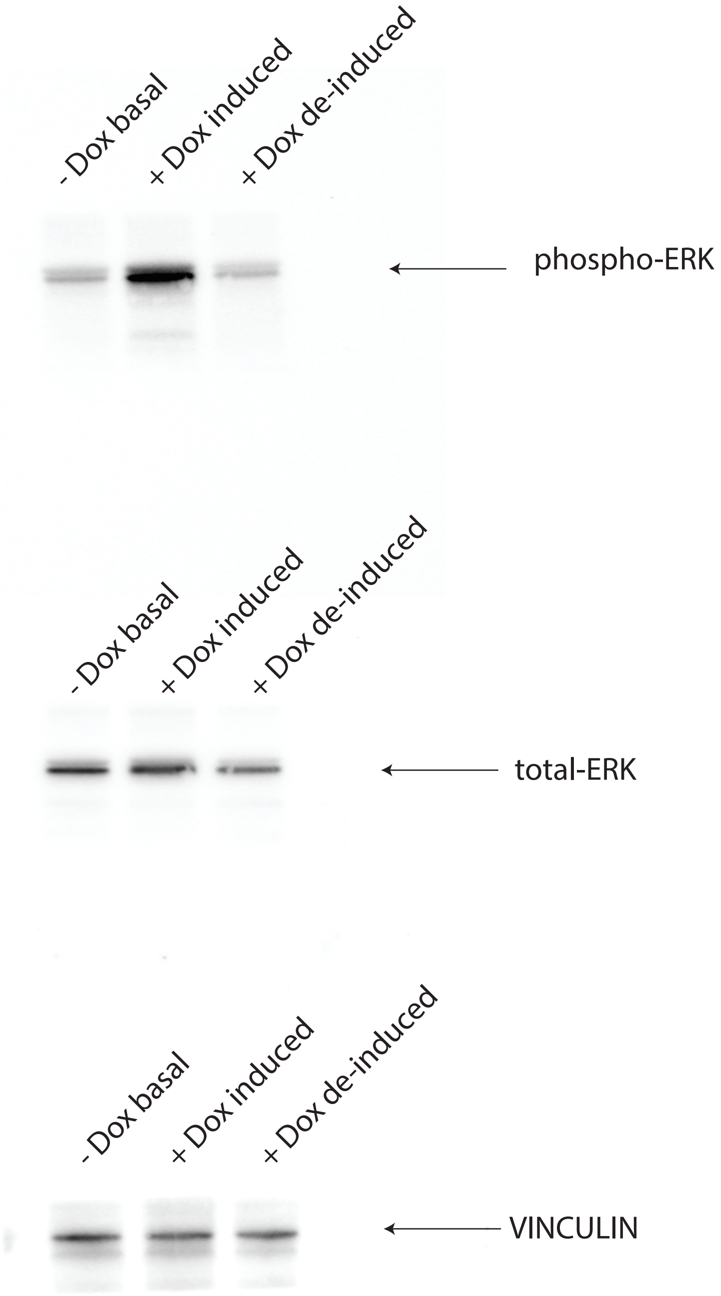
- Dox basal
+ Dox induced
+ Dox de-induced
phospho-ERK
- Dox basal
+ Dox induced
+ Dox de-induced
total-ERK
- Dox basal
+ Dox induced
+ Dox de-induced
VINCULIN

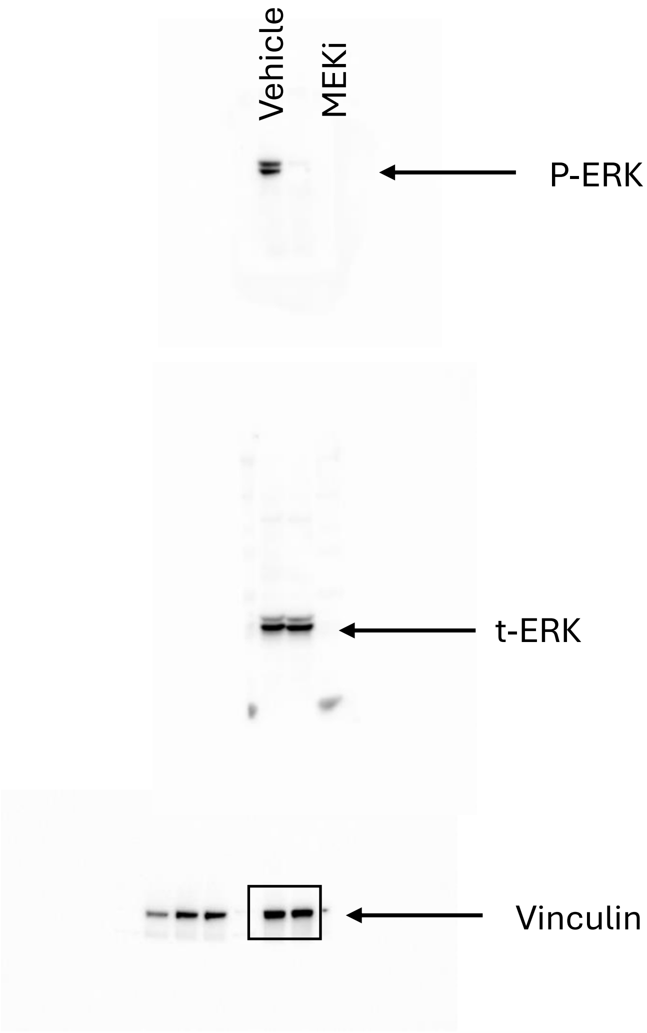
Vehicle
MEKi
P-ERK
t-ERK
Vinculin

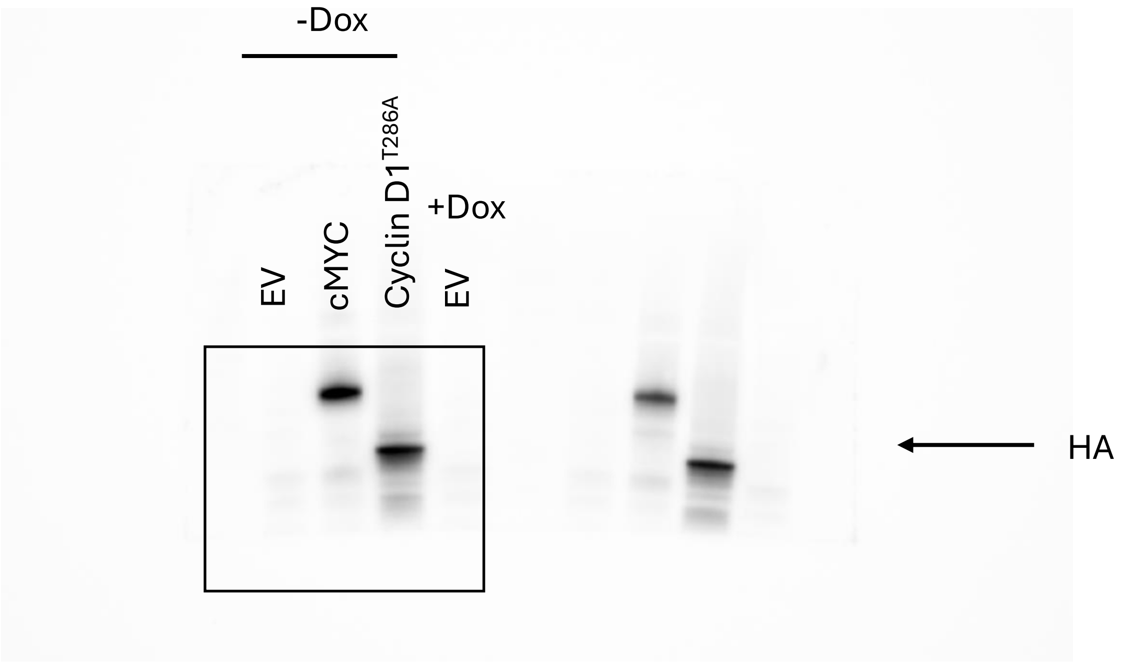
-Dox
EV
cMYC
Cyclin D1T286A
+Dox
EV
HA

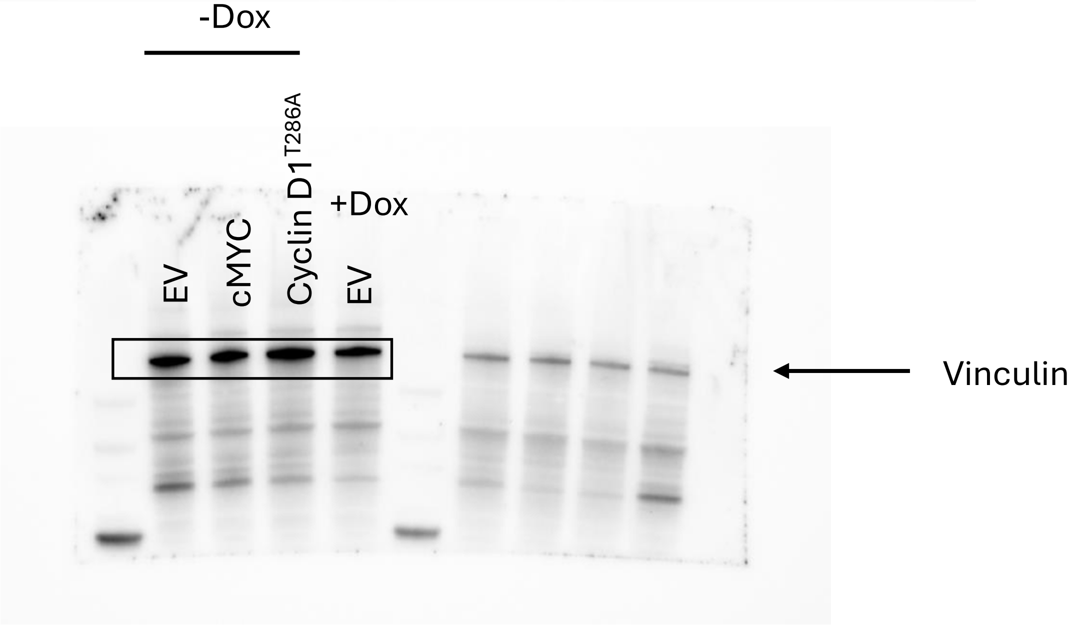
-Dox
EV
cMYC
Cyclin D1T286A
+Dox
EV
Vinculin

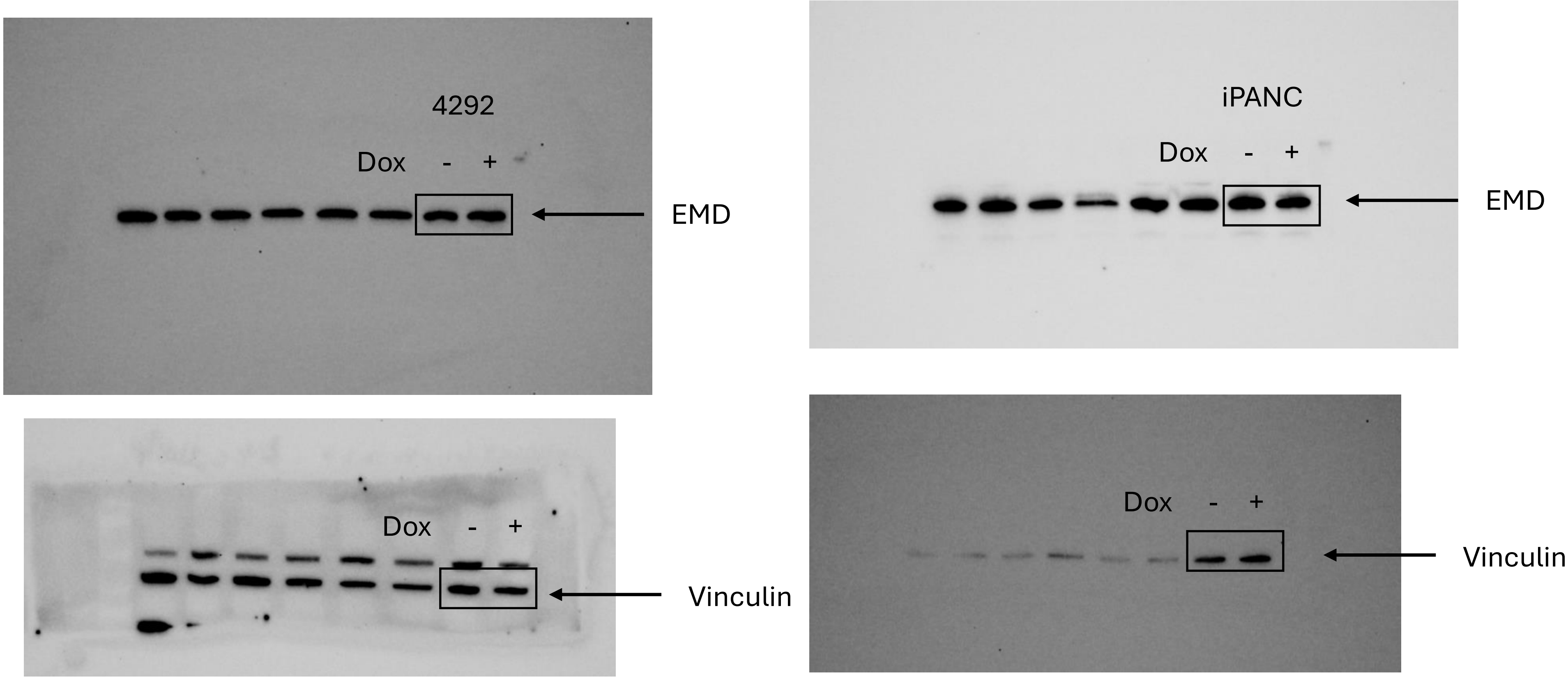
4292
Dox - +
EMD
Dox - +
Vinculin
iPANC
Dox - +
EMD
Dox - +
Vinculin

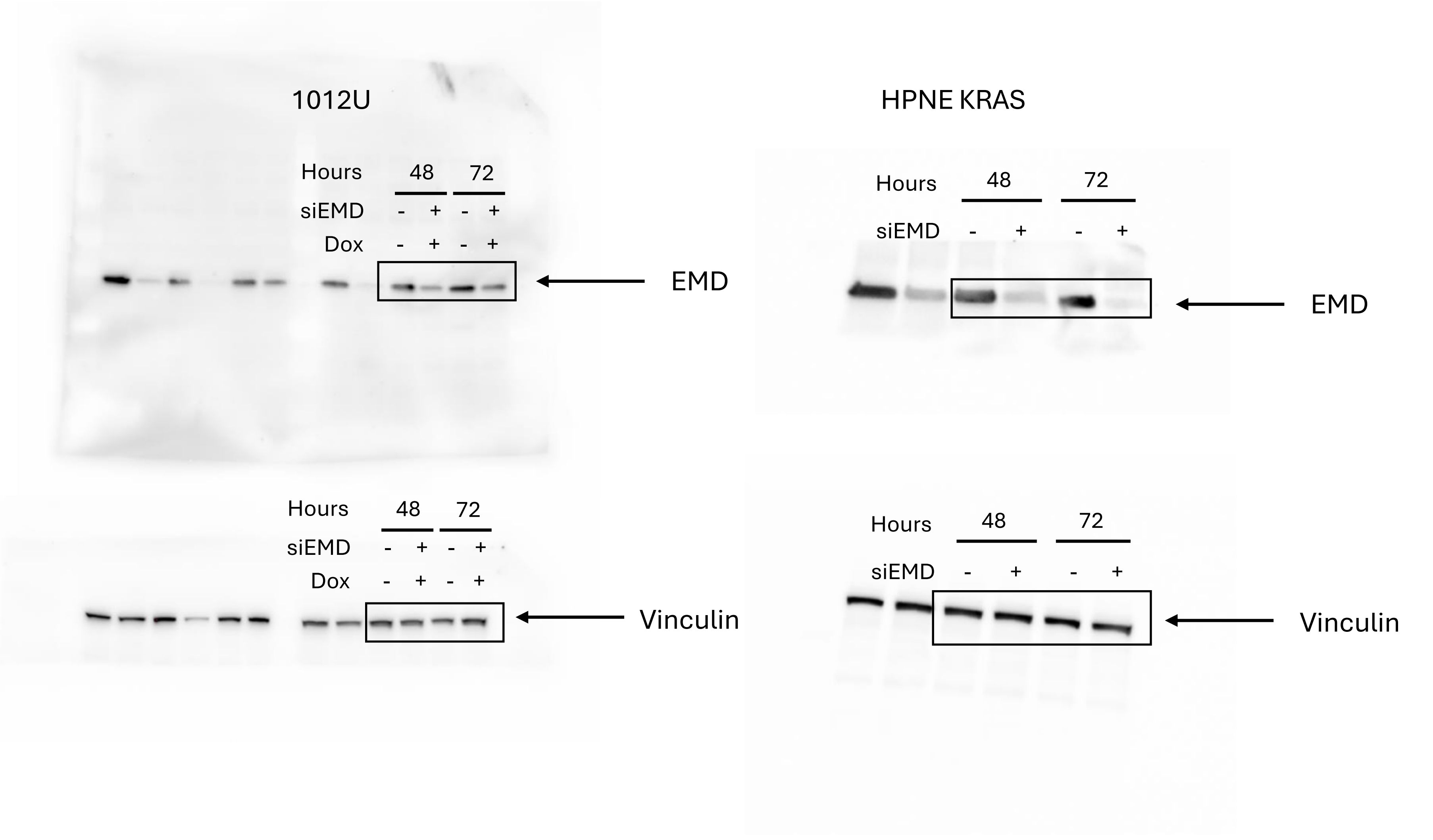
1012U
HPNE KRAS
Hours 48 72
siEMD - + - +
Dox - + - +
EMD
Hours 48 72
siEMD - + - +
Dox - + - +
Vinculin
Hours 48 72
siEMD - + - +
EMD
Hours 48 72
siEMD - + - +
Vinculin

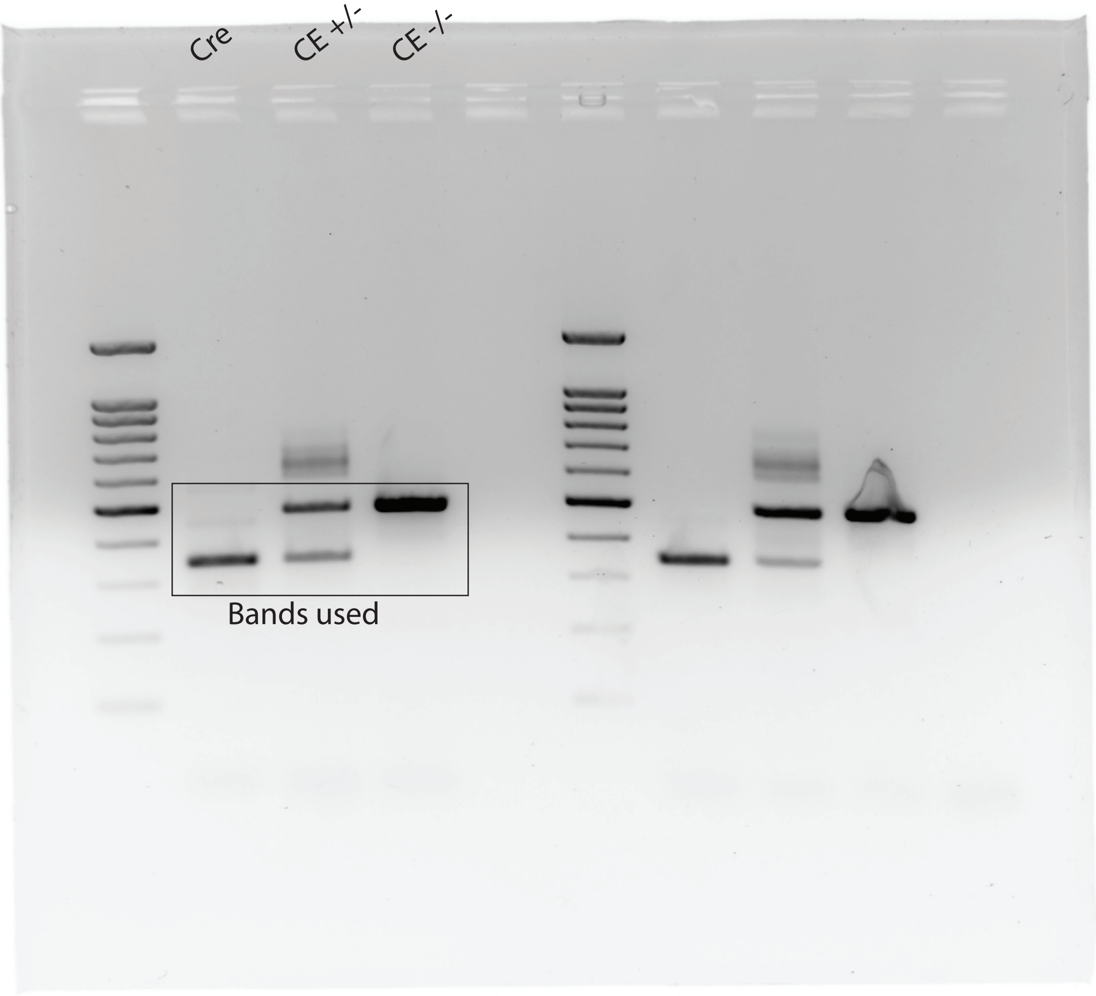
Cre
CE +/-
CE -/-
Bands used
